# Three Strategies Enhance the Bionic Coati Optimization Algorithm for Global Optimization and Feature Selection Problems

**DOI:** 10.3390/biomimetics10060380

**Published:** 2025-06-07

**Authors:** Qingzheng Cao, Shuqi Yuan, Yi Fang

**Affiliations:** 1 School of Mechanical Engineering, Hunan Institute of Science and Technology, Yueyang 414006, China; caoqingzheng2024@163.com; 2 School of Data Science and Ecommerce, Henan University of Economics and Law, Zhengzhou 450016, China; 202334170617@stu.huel.edu.cn; 3 School of Electronic Information and Electrical Engineering, Shanghai Jiao Tong University, Shanghai 200240, China

**Keywords:** adaptive search strategy, balancing factor, centroid guidance strategy, bionic coati optimization algorithm, feature selection

## Abstract

With the advancement of industrial digitization, utilizing large datasets for model training to boost performance is a pivotal technical approach for industry progress. However, raw training datasets often contain abundant redundant features, which increase model training’s computational cost and impair generalization ability. To tackle this, this study proposes the bionic ABCCOA algorithm, an enhanced version of the bionic Coati Optimization Algorithm (COA), to improve redundant feature elimination in datasets. To address the bionic COA’s inadequate global search performance in feature selection (FS) problems, leading to lower classification accuracy, an adaptive search strategy is introduced. This strategy combines individual learning capability and the learnability of disparities, enhancing global exploration. For the imbalance between the exploration and exploitation phases in the bionic COA algorithm when solving FS problems, which often traps it in suboptimal feature subsets, a balancing factor is proposed. By integrating phase control and dynamic adjustability, a good balance between the two phases is achieved, reducing the likelihood of getting stuck in suboptimal subsets. Additionally, to counter the bionic COA’s insufficient local exploitation performance in FS problems, increasing classification error rates, a centroid guidance strategy is presented. By combining population centroid guidance and fractional-order historical memory, the algorithm lowers the classification error rate of feature subsets and speeds up convergence. The bionic ABCCOA algorithm was tested on the CEC2020 test functions and engineering problem, achieving an over 90% optimization success rate and faster convergence, confirming its efficiency. Applied to 27 FS problems, it outperformed comparative algorithms in best, average, and worst fitness function values, classification accuracy, feature subset size, and running time, proving it an efficient and robust FS algorithm.

## 1. Introduction

Advancements in the field of Artificial Intelligence (AI) have spurred developments across various domains, including education, healthcare, and manufacturing [1,2,3,4,5,6,7,8,9,10]. Currently, large-scale model technologies within AI have emerged as a pivotal focal point [11,12,13,14,15]. Leveraging data for model training to enhance predictive and inferential performance has become a crucial technical approach, driving progress in these industries [16,17,18,19,20,21,22,23,24,25]. Unfortunately, however, the raw datasets employed for model training often contain a substantial number of redundant features, which diminishes the interpretability of the data and incurs computational costs while compromising the model’s generalization ability [26,27,28,29,30]. To enhance the interpretability and generalization capacity of models, it is typically necessary to eliminate redundant features within datasets, a process known as feature dimensionality reduction [31,32,33,34,35]. This not only improves model interpretability but also liberates additional computational resources. Assuming the original dataset comprises *M* feature information units, the task of feature dimensionality reduction involves searching through 2*^M^* possible feature subset combinations, which constitutes an NP-hard problem [36,37,38]. To reduce the computational cost associated with the feature subset search process, employing bionic-inspired feature selection methods to address this NP-hard problem has been demonstrated to be more appropriate [39,40,41,42,43,44]. Consequently, the objective of this paper is to propose a robust bionic-inspired feature selection (FS) method with efficient search capabilities. The aim is to leverage the computational simplicity of bionic-inspired algorithms to cut down on the computational expenses during the data denoising process, effectively eliminate redundant features, and enhance the interpretability and generalization ability of model training.

Currently, there are two primary approaches to FS: the filter method [45] and the wrapper method [46]. The filter method evaluates the significance of each feature independently by assessing statistical measures such as correlation [47], information gain [48], variance [49], and the chi-square test [50], among others. This enables the selection of an appropriate feature subset combination to represent the original dataset. However, this approach is prone to overlooking interactions among features [51], potentially leading to the exclusion of important features and a subsequent decline in classification accuracy. In contrast, the wrapper method is model performance-centric. It iteratively selects feature subsets, which are then incorporated into the model training process. The current feature subset is evaluated using algorithms such as K-Nearest Neighbors (KNN) [52], neural networks [53], and Support Vector Machines (SVM) [54]. Features are either removed or added to obtain the optimal feature subset that best represents the entire dataset, thereby enhancing classification accuracy. The primary advantage of this method lies in its consideration of interactions among features, significantly improving the model’s interpretability and substantially increasing the classification accuracy of the feature subset [41]. Compared to the filter method, the wrapper method better accounts for interactions among features and achieves higher classification accuracy for feature subsets. However, the wrapper method’s computational cost is elevated due to the need to exhaustively search through a vast number of feature subset combinations. Meta-heuristic algorithms derived from bionic simulations of natural organisms can significantly reduce the computational cost of traversing feature subsets while effectively enhancing classification accuracy, owing to their structural flexibility and simplicity [55].

Given the advantage of bionic algorithms in terms of low computational costs, numerous bionic-based FS methods have been proposed by current scholars to address FS problems, aiming to effectively eliminate redundant features while enhancing classification accuracy. For instance, Askr et al. [56] introduced a bionic algorithm named Binary Enhanced Golden Jackal Optimization (BEGJO) to improve classification accuracy in feature selection for high-dimensional datasets. They enhanced the original bionic Golden Jackal Optimization (GJO) algorithm by incorporating Copula entropy and four enhanced search strategies. Experimental results on 15 FS problems demonstrated bionic BEGJO’s superior performance in terms of classification accuracy and feature dimensionality, proving its effectiveness in high-dimensional feature selection. However, its drawback lies in its runtime ranking fourth, indicating potential for improvement. Abdel-Salam et al. [57] proposed an adaptive chaotic version of the bionic RIME algorithm, termed bionic ACRIME, which integrates four improvement strategies to enhance the algorithm’s FS performance. Subsequently, bionic ACRIME was applied to solve FS problems across 14 different datasets. Experimental results indicated that the proposed bionic algorithm effectively located the optimal feature subsets, improved classification accuracy, and eliminated redundant features. Nevertheless, this work still exhibits certain performance limitations when addressing high-dimensional FS problems and does not comprehensively consider runtime. Tijjani et al. [58] introduced a novel bionic Particle Swarm Optimization (PSO)-based FS method called bionic EBPSO, which enhances feature selection performance by incorporating a dimensionality reduction mechanism and a position update mechanism. Experimental results demonstrated its ability to effectively reduce feature dimensionality and runtime while improving classification accuracy. However, the proposed bionic method still has some shortcomings when dealing with high-dimensional FS problems, such as an imbalance between local and global searches, which increases the risk of becoming trapped in locally optimal feature subsets. Fang et al. [59] proposed a bionic genetic algorithm-based feature selection method to reduce redundant features in ICS data. The experimental results showed that, through a feature ranking fusion mechanism and a growth tree clustering idea, the method achieved effective feature selection for ICS data, leading to higher ICS processing performance. Despite its good performance, the issue of runtime has not been addressed. Ye et al. [60] proposed an improved bionic Hybrid Breeding Optimization (HBO) algorithm, termed bionic CCI-HBO, by introducing Lévy flights, elite opposition-based learning strategies, and cooperative co-evolution to enhance the algorithm’s FS performance. Experimental results indicated that bionic CCI-HBO outperformed over 90% of the FS problems, but it did not show a significant advantage in classification accuracy for high-dimensional FS problems. Wang et al. [61] proposed a role-oriented bionic Binary Grey Wolf Optimizer (BFLGWO) for feature selection, which enhances FS performance by incorporating a V-shaped linear transfer function and a role-oriented strategy. Experimental results demonstrated that the method excels in balancing exploitation and exploration and is competitive in feature selection tasks. Although bionic BFLGWO outperforms other bionic algorithms in terms of the number of selected features and classification accuracy, it has limitations in terms of time consumption. Houssein et al. [62] proposed an improved bionic mSTOA algorithm that optimizes FS performance by balancing exploration and exploitation, adaptive parameter control, and population reduction strategies. Experimental results on nine FS problems showed that it achieved the highest classification accuracy on over 89.5% of the problems. However, its limitation is that the tested FS problems are all in low-dimensional spaces, failing to represent high-dimensional FS performance. It may still exhibit performance deficiencies when addressing high-dimensional FS problems, and runtime has not been fully considered.

The aforementioned related works have demonstrated that metaheuristic-based bionic FS methods possess powerful capabilities for dataset dimensionality reduction, effectively eliminating redundant features and enhancing classification accuracy. However, as the dimensionality and scale of FS problems increase, existing bionic FS methods encounter issues such as low classification accuracy, an inability to eliminate redundant features, suboptimal runtime, and insufficient stability. The primary cause of these problems lies in the exponential growth of feature subset combinations that algorithms must search through as the dimensionality of FS problems rises. Due to limitations in their global exploration and local exploitation capabilities, bionic algorithms are unable to thoroughly traverse the feature space, thereby constraining classification accuracy. Additionally, most existing bionic works fail to achieve a good balance across multiple metrics, such as classification accuracy, feature subset size, runtime, stability, and convergence speed, when solving high-dimensional FS problems. Although they may excel in a specific metric, performance limitations in other metrics can compromise the bionic algorithm’s reliability. Consequently, these facts motivate us to propose a robust bionic FS algorithm that comprehensively considers multiple performance metrics to alleviate the problems of reduced classification accuracy and limited runtime caused by becoming trapped in locally suboptimal feature subsets in high-dimensional FS problems. Fortunately, the bionic Coati Optimization Algorithm (COA) has been validated as a robust tool with efficient optimization performance [63], and it has been applied in various fields, demonstrating strong application scalability. To date, however, no relevant researchers have applied bionic COA to solve FS problems. Therefore, this paper applies bionic COA to address FS problems to improve their solution performance while also filling a research gap in the bionic COA algorithm. Furthermore, considering the gradual increase in the dimensionality of current FS problems, the original bionic COA may suffer from insufficient global search and local exploitation capabilities during the solution process. To further enhance the algorithm’s FS performance, this paper proposes an enhanced bionic COA (ABCCOA) by integrating an adaptive search strategy, a balancing factor, and a centroid guidance strategy into the original bionic COA. Tests conducted on 27 FS problems confirm that the proposed bionic ABCCOA-based FS method exhibits significant advantages in terms of classification accuracy, stability, and runtime and can be considered a promising bionic FS approach. The main contributions of this paper are as follows:

- An adaptive search strategy is proposed, which integrates individual learning capabilities and the learnability of disparities, effectively enhancing the algorithm’s global exploration capabilities.
- A balancing factor is introduced, incorporating phase-based and dynamically adjustable characteristics, to achieve a well-balanced interplay between the exploration and exploitation phases.
- A centroid guidance strategy is devised, which combines the concept of centroid guidance with the utilization of fractional-order historical memory, thereby improving the algorithm’s exploitation capabilities.
- By integrating the aforementioned three strategies, the bionic ABCCOA algorithm is formulated. Experimental results on 27 FS problems demonstrate its superiority in terms of classification accuracy, stability, and runtime. This confirms that bionic ABCCOA is a promising bionic FS method.

The subsequent work plan for this paper is outlined as follows: In Section 2, the concept of the bionic COA is introduced. Section 3 proposes the bionic ABCCOA algorithm by integrating three learning strategies into the bionic COA framework. Section 4 validates the robust optimization performance of bionic ABCCOA through experiments conducted on the CEC2020 benchmark functions and engineering problems. Section 5 applies bionic ABCCOA to solve 27 FS problems, confirming its promise as an effective FS method. Finally, Section 6 provides a summary of the work presented in this paper and outlines the future research directions.

## 2. Mathematical Model of Coati Optimization Algorithm

In this section, the primary focus is on introducing the natural inspiration and mathematical model of the COA. The COA is a swarm intelligence optimization algorithm primarily formulated by simulating the attacking and escaping behaviors of coatis [63]. Specifically, the attacking behavior in the algorithm mainly simulates the global exploration phase of the COA, while the escaping behavior predominantly models the local exploitation phase. Additionally, when solving optimization problems using the COA, it is necessary to initialize a set of candidate solutions to form the initial population for subsequent iterative refinement. This process is referred to as the population initialization phase. Following this, the initial population undergoes updates in both the global search phase and the local exploitation phase to enhance the quality of the candidate solutions. In summary, the COA mainly consists of three phases: population initialization, global exploration, and local exploitation. A detailed description of each phase will be provided in the following subsections.

### 2.1. Population Initialization Phase

In this section, the focus is on introducing the population initialization phase of the COA. When utilizing the COA to solve optimization problems, the first step involves initializing a population comprising *N* individuals for subsequent algorithmic iterations. Each individual within this population represents a potential solution to the optimization problem and is characterized as a vector of size 1 × *D*, where *D* denotes the number of variables in the problem to be optimized. Under the aforementioned definitions, the initial population is computed using Equation (1).

(1) $$X={\left[\begin{array}{c} X_{1}\\ \vdots \\ X_{i}\\ \vdots \\ X_{N} \end{array}\right]}_{N\times 1}={\left[\begin{array}{ccccc} x_{1,1} & \ldots & x_{1,j} & \ldots & x_{1,D}\\ \vdots & \ddots & \vdots & \ddots & \vdots \\ x_{i,1} & \ldots & x_{i,j} & \ldots & x_{i,D}\\ \vdots & \ddots & \vdots & \ddots & \vdots \\ x_{N,1} & \ldots & x_{N,j} & \ldots & x_{N,D} \end{array}\right]}_{N\times D}$$

where X represents the initialized population, $X_{i}$ denotes the *ith* individual within the population, and $x_{i,j}$ signifies the value of the *jth* dimension for the *ith* individual. Specifically, $x_{i,j}$ is calculated using Equation (2).

(2) $$x_{i,j}=lb_{j}+rand\cdot (ub_{j}-lb_{j}),\;i=1,2,\ldots ,\;N,\;j=1,2,\ldots ,D.$$

where *rand* represents a random number within the interval [0, 1], while $ub_{j}$ and $lb_{j}$ denote the *jth* dimensional values of the upper and lower bounds, respectively, for the optimization problem to be solved. Subsequently, each individual is evaluated using its fitness function value to characterize its problem-solving capability. For minimization optimization problems, a smaller fitness function value indicates that the corresponding individual possesses a higher quality of problem-solving. The fitness function value corresponding to each individual is represented using Equation (3).

(3) $$F={\left[\begin{array}{c} F_{1}\\ \vdots \\ F_{i}\\ \vdots \\ F_{N} \end{array}\right]}_{N\times 1}={\left[\begin{array}{c} F(X_{1})\\ \vdots \\ F(X_{i})\\ \vdots \\ F(X_{N}) \end{array}\right]}_{N\times 1}$$

where F denotes the vector of fitness function values for all individuals in the population, $F_{i}$ represents the fitness function value corresponding to the *ith* individual, and F(·) signifies the computational function for the fitness function value. After the population initialization is completed, it is necessary to optimize the quality of individuals within the population through the global exploration phase and the local exploitation phase. In the following subsections, the global exploration phase and the local exploitation phase of the COA algorithm will be introduced in detail.

### 2.2. Global Exploration Phase

**Figure 1:**
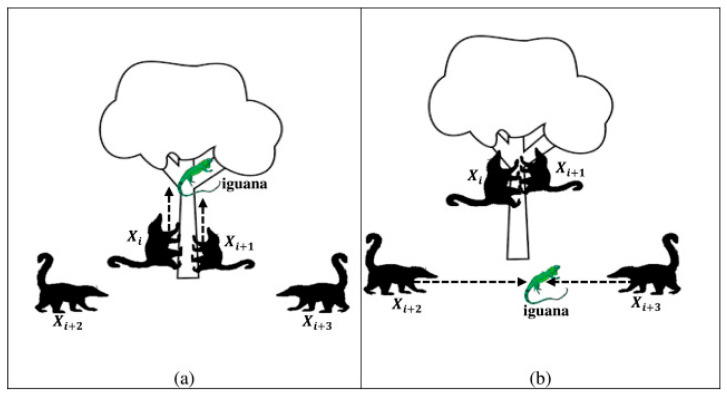
Schematic diagram of attack phase. (**a**). Launch an attack on the tree. (**b**). Launch an attack on the ground.

In this section, the global exploration phase of the COA will be introduced in detail. This phase is primarily inspired by the attacking behavior of coatis towards iguanas. In this behavior, coatis climb trees to intimidate iguanas, causing them to fall to the ground. Subsequently, the coatis on the ground attempt to attack the iguanas, enabling them to move to various positions within the search space. As illustrated in Figure 1, this attacking behavior effectively mirrors the global search characteristic of the algorithm, allowing it to explore a wider range of potential optimal solution regions when solving optimization problems. In this context, we assume that the position of the iguanas represents the optimal individual within the population. Half of the coatis climb trees to launch attacks on the iguanas, while the other half remain on the ground to target the fallen iguanas. Based on this assumption, the behavior of coatis climbing trees to attack the iguanas is mathematically modeled using Equation (4).

(4) $$x_{i,j}^{P1}=x_{i,j}+rand\cdot \left(Iguana_{j}-I\cdot x_{i,j}\right),\;i=1,2,\ldots ,\left\lfloor \frac{N}{2}\right\rfloor ,\;j=1,2,\ldots ,D.$$

where $x_{i,j}^{P1}$ denotes the new state of the *jth* dimension of the *ith* individual after simulating the attacking behavior. $Iguana_{j}$ represents the value of the *jth* dimension of the best individual, and *I* is a randomly selected constant from the set {1,2}. After the iguanas fall to random positions on the ground, the coatis on the ground launch attacks on them. This behavior is mathematically simulated by Equation (5).

(5) $$\begin{array}{l} x_{i,j}^{P1}=\left\{\begin{array}{ll} x_{i,j}+rand\cdot \left(Iguana_{j}^{G}-I\cdot x_{i,j}\right), & if\;F_{Iguana^{G}}<F_{i}\\ x_{i,j}+rand\cdot \left(x_{i,j}-Iguana_{j}^{G}\right), & else \end{array}\right.,\\ i=\left\lfloor \frac{N}{2}\right\rfloor +1,\left\lfloor \frac{N}{2}\right\rfloor +2,\ldots ,N,\;j=1,2,\ldots ,D. \end{array}$$

where $F_{i}$ represents the fitness function value of the *ith* individual, and $\;F_{Iguana^{G}}$ denotes the fitness function value of the iguanas that have fallen to the ground. $Iguana_{j}^{G}$ signifies the value of the *jth* dimension of the position of the iguanas that have fallen to the ground; this is calculated using Equation (6).

(6) $$Iguana_{j}^{G}=lb_{j}+rand\cdot (ub_{j}-lb_{j}),\;j=1,2,\ldots ,D.$$

Subsequently, the new state generated by an individual during the attacking behavior is retained using Equation (7).

(7) $$X_{i}=\left\{\begin{array}{ll} X_{i}^{P1}, & if\;F_{i}^{P1}<F_{i}\\ X_{i}, & else \end{array}\right.$$

where $X_{i}^{P1}$ denotes the new individual state generated by the *ith* individual through the simulation of the attacking behavior, and $F_{i}^{P1}$ represents the fitness function value corresponding to the individual $X_{i}$. By simulating the attacking behavior of coatis, the global exploration capability of the COA algorithm is ensured. In the following subsection, the local exploitation phase of the COA algorithm will be introduced in detail.

### 2.3. Local Exploitation Phase 

**Figure 2:**
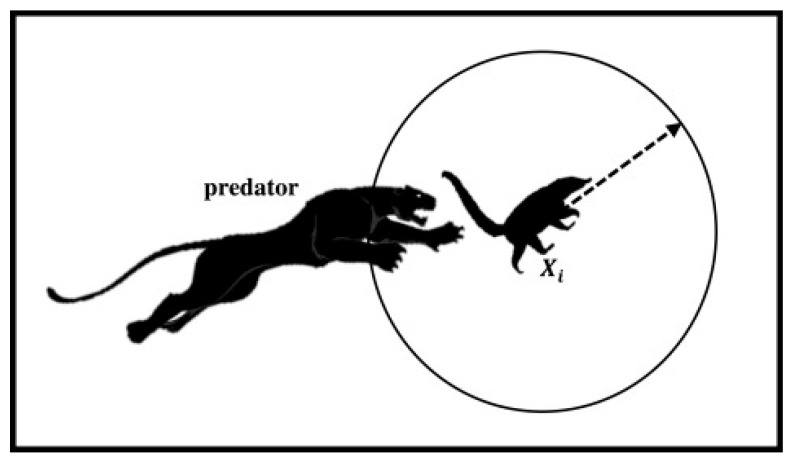
Principle diagram of escape phase.

In this section, a detailed description is provided regarding the local exploitation phase of the COA algorithm. During this phase, the focus is on simulating the escape behavior of coatis when confronted with predators. When coatis are subjected to attacks from predators, they promptly flee within a designated safe zone to ensure their safety. As illustrated in Figure 2, by simulating this behavior, the algorithm’s local exploitation performance is effectively guaranteed, enabling it to further explore and exploit the identified regions of potential optimal solutions. Specifically, Equation (8) is utilized to simulate the escape behavior of coatis.

(8) $$x_{i,j}^{P2}=x_{i,j}+(1-2r)\cdot \left(lb_{j}^{local}+r\cdot (ub_{j}^{local}-lb_{j}^{local})\right),i=1,2,\ldots ,N,\;j=1,2,\ldots ,D.$$

where $x_{i,j}^{P2}$ represents the new state of the *jth* dimension for the *ith* individual after simulating the escape behavior, *r* denotes a random number generated within the interval [0, 1], and both $lb_{j}^{local}$ and $ub_{j}^{local}$ are calculated using Equation (9).

(9) $$lb_{j}^{local}=\frac{lb_{j}}{t},\;ub_{j}^{local}=\frac{ub_{j}}{t},\;t=1,2,\ldots ,T.$$

where *t* represents the current iteration number of the algorithm, and *T* denotes the maximum number of iterations for the algorithm. Subsequently, the new state generated by an individual during the escape behavior is retained using Equation (10).

(10) $$X_{i}=\left\{\begin{array}{ll} X_{i}^{P2}, & if\;F_{i}^{P2}<F_{i}\\ X_{i}, & else \end{array}\right.$$

where $X_{i}^{P2}$ represents the new individual state generated by the *ith* individual after simulating the escape behavior, and $F_{i}^{P2}$ denotes the fitness function value corresponding to the individual $X_{i}^{P2}$. By simulating the escape behavior of coatis, the algorithm’s local exploitation capability is ensured, thereby guaranteeing the optimization precision. After introducing the population initialization phase, the global exploration phase, and the local exploitation phase involved in the COA algorithm, the next subsection will present the execution logic of the COA algorithm.

### 2.4. Execution Framework of COA Algorithm 

**Figure 3:**
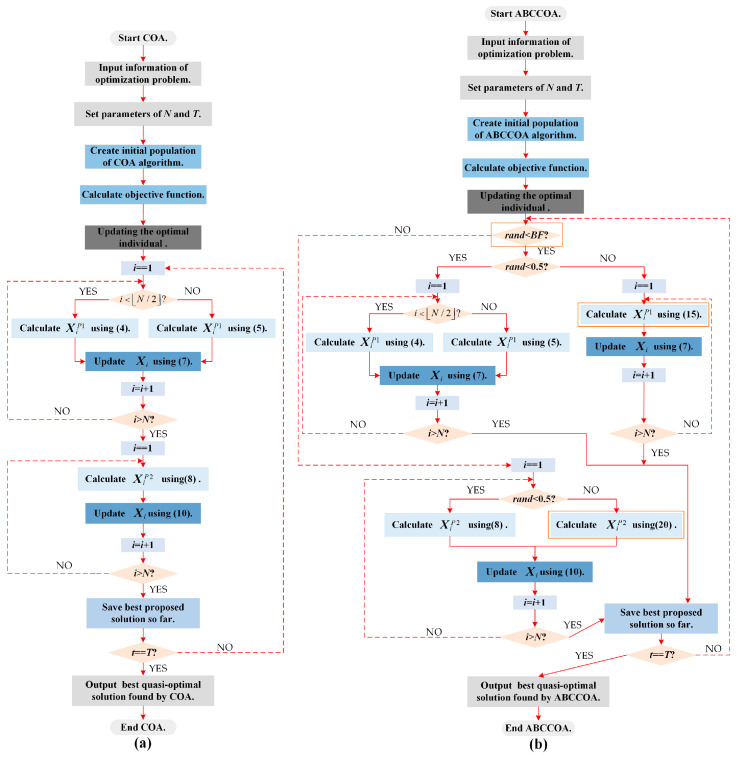
Algorithm execution flowchart: (**a**) COA algorithm; (**b**) ABCCOA algorithm.

In this section, the execution logic of the COA algorithm when solving optimization problems will be introduced. When the COA algorithm is applied to solve an optimization problem, it first initializes the population to generate a set of high-quality candidate solutions. Subsequently, through the synergistic effects of the global exploration phase and the local exploitation phase, the algorithm enhances the quality of individuals on a population-wide basis, thereby effectively addressing the optimization problem. The pseudo code for the COA algorithm is presented in Algorithm 1. Additionally, to facilitate an intuitive understanding of the algorithm’s execution logic, Figure 3a illustrates the flowchart of the algorithm’s execution.
**Algorithm 1:** Pseudo code of COA algorithm**Input:** Population size (*N*), Dimension (*D*), Upper bound (***ub***) and lower bound (***lb***), Maximum of iterations (*T*).**Output:** Global best solution (*X_best_*).1.  Initialize population based on Equation (1) and calculate the individual fitness function values of the population. 2.  ***for***
*t* = 1:*T* 3.    Calculate positions of iguanas using the globally best individual. 4.    Phase 1: The attack behavior of coatis (Global Exploration Phase) 5.    for $i=1:\left\lfloor N/2\right\rfloor$ 6.     for *j* = 1:*D* 7.       Calculate the *jth* dimensional new state of the *ith* individual based on Equation (4). 8.     end for 9.    end for 10.  for $i=1+\left\lfloor N/2\right\rfloor :N$ 11.    for *j* = 1:*D* 12.      Calculate the *jth* dimensional new state of the *ith* individual based on Equation (5). 13.    end for 14.  end for 15.  Use Equation (7) to preserve the new state of individual $X_{i}$. 16.  Phase 2: The escape behavior of coatis (Local Exploitation Phase) 17.  for *i* = 1:*N*
18.    for *j* = 1:*D* 19.      Calculate the *jth* dimensional new state of the *ith* individual based on Equation (8). 20.    end for 21.  end for 22.  Use Equation (10) to preserve the new state of individual $X_{i}$. 23.  Save the global best solution *X_best_*. 24. end for 25. Output the global best solution *X_best_* obtained by solving the optimization problem using the COA algorithm.

## 3. The Proposal of ABCCOA Algorithm

The original COA algorithm suffers from insufficient global exploration capability, inadequate local exploitation capability, and an imbalance between the exploration and exploitation phases when solving FS problems. These issues lead to the algorithm easily becoming trapped in suboptimal feature subset combinations and experiencing local stagnation, particularly when addressing high-dimensional FS problems, thereby compromising the classification accuracy of feature subsets. To enhance the FS performance of the COA algorithm, this paper proposes an enhanced COA algorithm, termed ABCCOA, by integrating three learning strategies into the COA framework, thereby improving its FS performance from various perspectives. In the ABCCOA algorithm, firstly, an adaptive search strategy is introduced to address the deficiency in global search performance of the COA algorithm when solving FS problems due to the exponential increase in the number of feature subset combinations, which, in turn, reduces classification accuracy. This strategy facilitates learning from the disparities between different types of individuals, taking into account both the individual’s own learning capacity and the learnability of each disparity group. It effectively enhances the population diversity during the iterative process, thereby strengthening the algorithm’s global exploration capability and improving the classification accuracy of feature subsets. Secondly, to tackle the issue of the COA algorithm’s tendency to become trapped in suboptimal feature subset combinations due to an imbalance between the exploration and exploitation phases when solving FS problems, a balancing factor is proposed to better control the global exploration and local exploitation phases. In the proposed balancing factor, the phase is controlled through sine and cosine functions, while arcsine and arccosine functions are also incorporated for dynamic adjustment. This ensures that the algorithm maintains higher global exploration capability while also guaranteeing a certain level of exploitation capability during the early iterations, contributing to improved convergence speed and accuracy. Meanwhile, during the later iterations, the algorithm exhibits higher local exploitation capability while still ensuring a certain level of global search capability, reducing the probability of the algorithm becoming trapped in suboptimal FS subsets. Finally, to address the issue of increased classification error rates resulting from insufficient local exploitation capability of the COA algorithm when solving FS problems, a centroid guidance strategy is proposed to enhance the algorithm’s local exploitation capability. In this strategy, guidance is provided by leveraging the centroids of individuals from the previous four generations of the population, combined with the fractional-order historical memory effect. This approach not only reduces the classification error rate of FS subsets but also accelerates the convergence speed of the algorithm. In the following sections, we will elaborate on the mathematical concepts of the adaptive learning strategy, the balancing factor, and the centroid guidance strategy, as well as the execution logic of the ABCCOA algorithm.

### 3.1. Adaptive Search Strategy

The original COA algorithm exhibits insufficient global exploration capability when addressing complex FS problems, primarily due to the increase in the number of features within the original dataset. This deficiency leads to reduced population diversity, hindering the algorithm’s ability to locate regions containing potentially optimal FS subset combinations and subsequently decreasing the classification accuracy of the subsets. To mitigate this issue, there is an urgent need to propose a search strategy with efficient exploratory capabilities. Zhang et al. [64] have indicated that learning by individuals from the gaps between different types of individuals within the population can enhance the algorithm’s global exploration capability. Inspired by this, this section proposes an adaptive search strategy to augment the algorithm’s global exploratory performance. In the adaptive search strategy, individuals primarily learn from four sets of individual gaps: the gap between the globally best individual and better individuals ($Gap_{1}$), the gap between the globally best individual and worse individuals ($Gap_{2}$), the gap between better and worse individuals ($Gap_{3}$), and the gap between two randomly selected individuals ($Gap_{4}$). These four sets of gaps are calculated using Equation (11).

(11) $$\left\{\begin{array}{l} Gap_{1}=X_{best}-X_{better}\\ Gap_{2}=X_{best}-X_{worse}\\ Gap_{3}=X_{better}-X_{worse}\\ Gap_{4}=X_{rand1}-X_{rand2} \end{array}\right.$$

where $X_{best}$ denotes the globally best individual, and $X_{better}$ represents a better individual, which is defined as a randomly selected individual from the set of the top five individuals with the smallest fitness function values within the population. $X_{worse}$ denotes a worse individual, defined as a randomly selected individual from the set of the top five individuals with the largest fitness function values within the population. $X_{rand1}$ and $X_{rand2}$ represent two distinct random individuals selected from the population. Moreover, the learnability of each gap of individual disparities also varies. We employed Equation (12) to calculate the learnability of each gap of disparities.

(12) $$LB_{k}=\frac{\left\|Gap_{k}\right\|}{\sum_{k=1}^{4}\left\|Gap_{k}\right\|},\;(k=1,2,3,4)$$

where $LB_{k}$ denotes the learnability of the *kth* gap of disparities. As can be observed from the equation, the greater the disparity between individuals, the higher the learnability of this gap of disparities. This is because a larger disparity implies a greater amount of information embedded within this gap. Furthermore, individuals with different characteristics exhibit varying learning capacities. We utilized Equation (13) to calculate the learning capacity of each individual.

(13) $$LC_{i}=\frac{F_{i}}{F_{max}}$$

where $LC_{i}$ represents the learning capacity of the *ith* individual, $F_{i}$ denotes the fitness function value of the *ith* individual, and $F_{max}$ signifies the maximum fitness function value within the population. As can be inferred from the equation, individuals with smaller fitness function values possess lower learning capacities. This is primarily because a smaller fitness function value implies that the individual is of higher quality. Consequently, it is necessary to reduce the global exploration phase to preserve the individual’s quality and enhance the optimization precision, and vice versa. Based on the aforementioned definitions, Equation (14) is employed to calculate the learning process of the *ith* individual from the *kth* gap of disparities.

(14) $$KA_{k}=LC_{i}\cdot LB_{k}\cdot Gap_{k},\;(k=1,2,3,4)$$

where $KA_{k}$ represents the information acquired by the *ith* individual after learning from the *kth* gap of disparities. Subsequently, Equation (15) is utilized to compute the new state generated by the *ith* individual after learning from all four gaps of disparities.

(15) $$X_{i}^{P1}=X_{i}+KA_{1}+KA_{2}+KA_{3}+KA_{4}$$

**Figure 4:**
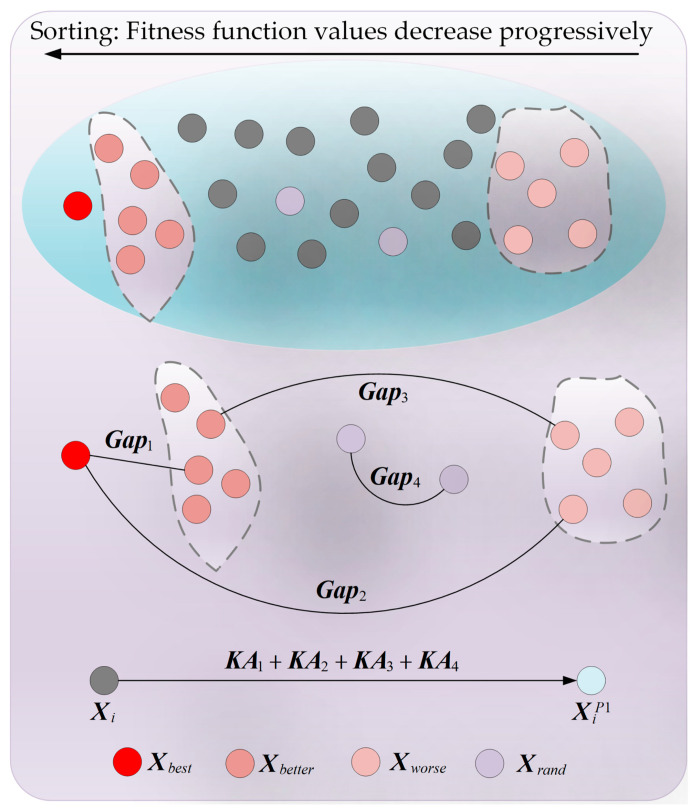
Schematic of adaptive search strategy.

Subsequently, Equation (7) is employed to retain the new state of the individual. To provide a more intuitive representation of the adaptive search strategy, Figure 4 visualizes the entire search process.

### 3.2. Balancing Factor

**Figure 5:**
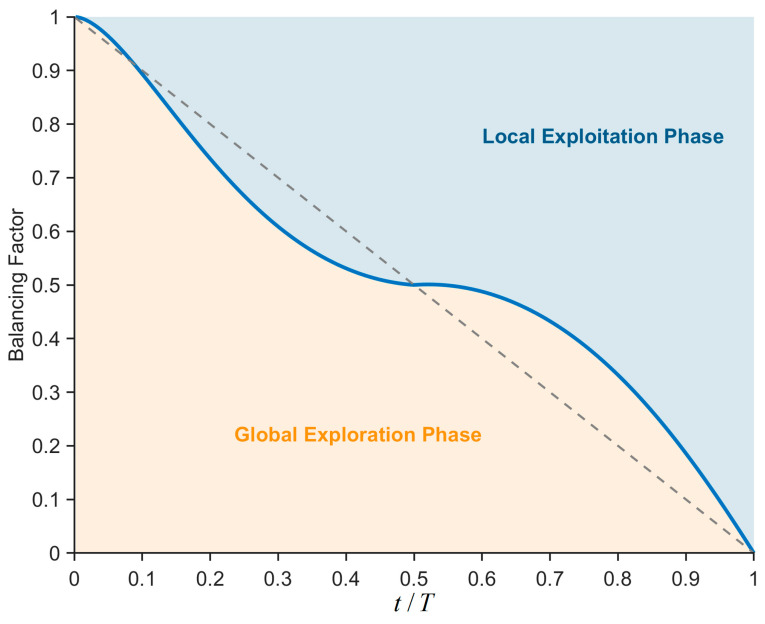
Schematic of balancing factor.

When the original COA algorithm is applied to solve feature selection (FS) problems, as the number of feature elements in the original dataset increases dramatically, the algorithm is required to search through feature subset combinations that grow exponentially in magnitude. This results in the COA algorithm lacking a proper balance between global exploration and local exploitation, making it prone to becoming trapped in locally suboptimal feature subset combinations and exacerbating the deterioration of classification error rates. To alleviate this issue, this section proposes a novel balancing factor to reasonably balance the global exploration phase and the local exploitation phase. This factor is primarily composed of trigonometric and inverse trigonometric functions. Specifically, the sine and cosine functions mainly control the phase of the balancing factor, while the arcsine and arccosine functions primarily provide dynamic adjustments to the balancing factor. This configuration enables the algorithm to possess higher global exploration capabilities while ensuring a certain level of exploitation capability during the early iterations, thereby enhancing the algorithm’s convergence speed and precision. Simultaneously, it ensures higher local exploitation performance during the later iterations while maintaining a certain degree of global search capability, reducing the probability of the algorithm becoming stuck in suboptimal FS subsets. Specifically, the phase adjustment term formed by the combination of the sine and cosine functions is expressed as Equation (16).

(16) $$pat={\left(sin(\pi \cdot t/2T)\right)}^{2}+0.5\cdot (1-cos(\pi \cdot t/T))$$

where *pat* denotes the phase adjustment term. The dynamic adjustment term, which is formed by combining the arcsine and arccosine functions, is expressed as Equation (17).

(17) $$dat=\frac{2}{\pi }\cdot (arcsin(\sqrt{t/T})+arccos(\sqrt{t/T}))$$

where *dat* represents the dynamic adjustment term, arcsin(·) denotes the arcsine operation, and arccos(·) signifies the arccosine operation. Subsequently, the phase adjustment term *pat* and the dynamic adjustment term *dat* are combined to form the balancing factor proposed in this section, which is expressed as Equation (18).

(18) $$BF=\left\{\begin{array}{ll} {\left(\frac{t}{T}\right)}^{dat\cdot pat} & if\;(\frac{t}{T})<0.5\\ 1-{\left(\frac{t}{T}\right)}^{dat\cdot pat} & else \end{array}\right.$$

where *BF* denotes the balancing factor, and its value decreases non-linearly as the number of iterations t increases. To better illustrate this decreasing process, Figure 5 visualizes the changes in *BF* with the growth of the number of iterations. As can be observed from the figure, during the early stages of iteration, the global exploration phase of the algorithm dominates. However, as iterations progress, the local exploitation phase takes precedence. It is noteworthy that, even in the later stages of iteration, the algorithm still retains a certain degree of global exploration capability. This enables the algorithm to enhance classification accuracy while simultaneously reducing the probability of becoming trapped in locally suboptimal FS subset combinations. In summary, the balancing factor proposed in this section facilitates a better equilibrium between the global exploration and local exploitation phases of the algorithm when addressing FS problems. This contributes to identifying superior FS subset combinations and mitigating the classification error rate.

### 3.3. Centroid Guidance Strategy

When the original COA algorithm is employed to solve FS problems after locating a potentially optimal FS subset combination region during the global exploration phase, the algorithm’s classification accuracy and convergence speed are compromised due to its inadequate local exploitation capabilities. To address this issue, this section proposes a centroid guidance strategy to enhance the algorithm’s local exploitation performance. In the proposed centroid guidance strategy, individuals are primarily guided by the centroid individuals from the current population and the centroids of the preceding three generations. Simultaneously, the fractional-order theory is incorporated to weigh the centroid individuals of populations from different generations, thereby endowing the centroid guidance strategy with adaptability and local exploitation properties. Specifically, the centroid individual within a population is computed using Equation (19).

(19) $$X_{cen}^{t}=\sum_{i=1}^{N}\frac{F_{max}^{t}-F_{i}^{t}+\varepsilon }{\sum_{j=1}^{N}\left(F_{max}^{t}-F_{j}^{t}+\varepsilon \right)}\cdot X_{i}^{t}$$

where $X_{cen}^{t}$ denotes the centroid individual of the population at the *tth* iteration, $F_{max}^{t}$ represents the maximum fitness function value within the population at the *tth* iteration, $F_{i}^{t}$ signifies the fitness function value of the *ith* individual at the *tth* iteration, ε is a very small constant introduced to ensure that the denominator of the equation does not equal zero, and $X_{i}^{t}$ indicates the information of the *ith* individual at the *tth* iteration. Subsequently, the centroid individuals of the population at the *tth*, (t−1)th, (t−2)th, and (t−3)th iterations are calculated using Equation (19), denoted as $X_{cen}^{t}$, $X_{cen}^{t-1}$, $X_{cen}^{t-2}$, and $X_{cen}^{t-3}$, respectively. Then, fractional-order weights are employed as the learning weights for these four centroid individuals to guide the *ith* individual, thereby forming the centroid guidance strategy, which is expressed as Equation (20).

(20) $$\begin{array}{c} X_{i}^{P2}=X_{i}^{t}+\frac{1}{1!}\cdot q\cdot (X_{cen}^{t}-X_{i}^{t})+\frac{1}{2!}\cdot q\cdot (1-q)\cdot (X_{cen}^{t-1}-X_{i}^{t})+\frac{1}{3!}\cdot q\cdot (1-q)\cdot (2-q)\cdot \\ \left(X_{cen}^{t-2}-X_{i}^{t})+\frac{1}{4!}\cdot q\cdot (1-q)\cdot (2-q)\cdot (3-q)\cdot (X_{cen}^{t-3}-X_{i}^{t}\right) \end{array}$$

where q represents the adaptive factor, which is utilized to control the probability of the guidance process occurring, and it is calculated using Equation (21).

(21) $$q=\frac{1}{1+e^{\left(\frac{t}{T}\right)}}\cdot \cos\left(2\cdot \pi \cdot \left(\frac{t}{T}\right)\right)$$

**Figure 6:**
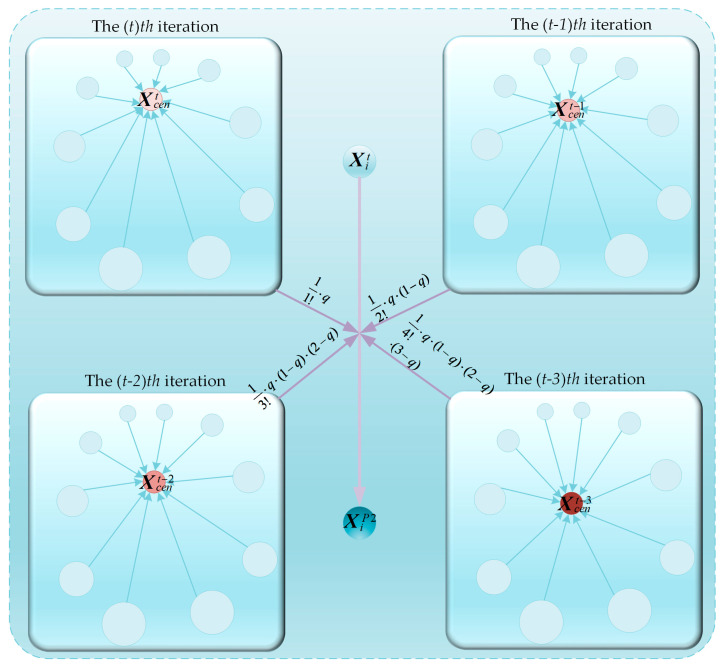
Schematic of centroid guidance strategy.

To provide a more intuitive illustration of the implementation process of the centroid guidance strategy, we have visualized it as shown in Figure 6. In the centroid guidance strategy, firstly, the concept of centroid individuals is introduced. These individuals, being the most representative within the population, are employed to guide the population’s evolution, offering significant advantages in local exploitation. Additionally, by integrating fractional-order theory, comprehensive consideration is given to the centroid individuals of four consecutive generations. This further enhances the algorithm’s local exploitation capabilities, enabling it to achieve faster convergence speeds and higher classification accuracy when solving complex FS problems.

### 3.4. Execution Framework of ABCCOA Algorithm 

To address the issues of inadequate global exploration capability, insufficient local exploitation capability, and imbalance between the global exploration and local exploitation phases in the COA when solving FS problems, this paper enhances its performance from various perspectives by integrating an adaptive search strategy, a balancing factor, and a centroid guidance strategy. These enhancements improve the algorithm’s global search and local exploitation capabilities while maintaining a balance between the global exploration and local exploitation phases, thereby further enhancing its classification accuracy. Consequently, this section primarily introduces the execution logic of the enhanced COA algorithm that incorporates these three learning strategies, referred to as the ABCCOA algorithm. Algorithm 2 presents the pseudo code for the execution of ABCCOA. Additionally, to visually demonstrate the execution logic of the ABCCOA algorithm, Figure 3b provides a flowchart of the ABCCOA algorithm.
**Algorithm 2:** Pseudo code of ABCCOA algorithm**Input:** Population size (N), Dimension (D), Upper bound (ub) and lower bound (lb), Maximum of iterations (T).**Output:** Global best solution ($X_{best}$).1.  Initialize population based on Equation (1) and calculate the individual fitness function values of the population. 2.  ***for*** t=1:T 3.    Calculate positions of iguanas using the globally best individual. 4.    Calculate Balancing Factor based on Equation (18). 5.    ***if*** rand<BF 6.     Phase 1: The attack behavior of coatis (Global Exploration Phase) 7.     ***if*** rand<0.5 8.      for $i=1:\left\lfloor N/2\right\rfloor$ 9.       for j=1:D 10.        Calculate the jth dimensional new state of the ith individual based on Equation (4). 11.     end for 12.   end for 13.   for $i=1+\left\lfloor N/2\right\rfloor :N$ 14.    for j=1:D 15.     Calculate the jth dimensional new state of the ith individual based on Equation (5). 16.    end for 17.   end for 18.  else 19.   for i=1:N 20.    Calculate the new state of the ith individual based on Equation (15). 21.   end for 22.  end if 23.  Use Equation (7) to preserve the new state of individual $X_{i}$. 24.  else 25.  Phase 2: The escape behavior of coatis (Local Exploitation Phase) 26.   ***if*** rand<0.5 27.   for i=1:N 28.    for j=1:D 29.     Calculate the jth dimensional new state of the ith individual based on Equation (8). 30.    end for 31.   end for 32.  else 33.   for i=1:N 34.    Calculate the new state of the ith individual based on Equation (20). 35.   end for 36.  end if 37.  end if 38.  Use Equation (10) to preserve the new state of individual $X_{i}$. 39.  Save the global best solution $X_{best}$. 40. end for 41. Output the global best solution $X_{best}$ obtained by solving the optimization problem using the COA algorithm.

## 4. Experimental Results and Discussion on CEC2020 and Real Problems

In this section, the primary focus is on evaluating the global optimization performance of the proposed ABCCOA algorithm. Specifically, tests are conducted using the CEC2020 benchmark function suite and five real engineering optimization problems, the details of which are outlined in Table 1. To ensure experimental fairness, the population size is set to 40 and the maximum number of function evaluations is capped at 40,000. Each experiment is independently replicated 30 times without repetition. Subsequently, the experimental results are subjected to comprehensive analyses, including population diversity analysis, exploration/exploitation balance analysis, fitness function value analysis, solution stability analysis, nonparametric statistical testing, and convergence analysis. These evaluations collectively assess the optimization performance of the ABCCOA algorithm. Additionally, to visually demonstrate ABCCOA’s optimization capabilities, we compare its performance against seven state-of-the-art optimization algorithms. The parameter configurations for these comparative algorithms are provided in Table 2. To ensure the reproducibility of the experiments, the hardware configuration employed was an Intel(R) Core (TM) Ultra 5 processor with 32 GB of RAM. The software environment consisted of MATLAB R2021b running on the Windows 11 operating system.

### 4.1. Strategies and Parameter Analysis

**Figure 7:**
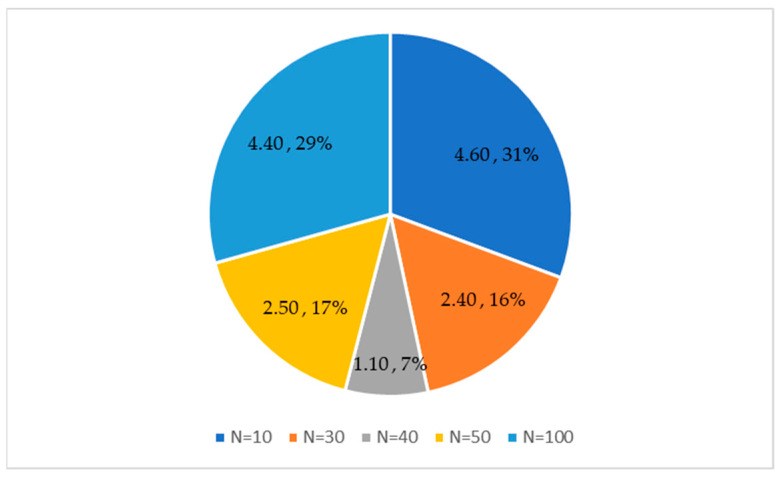
Average ranking of algorithms under different population sizes.

In this section, we primarily analyze the sensitivity of the algorithm with respect to population size and learning strategies. Specifically, experiments were conducted on the CEC2020 test functions. To select an appropriate population size that enhances the algorithm’s performance, we set the population sizes to 10, 30, 40, 50, and 100 for separate experimental runs. The experimental results are depicted in Figure 7. It can be observed that when the population size is 40, the algorithm achieves an average ranking of 1.10, securing the top position. This is primarily because a larger population tends to weaken the algorithm’s convergence tendency, while a smaller population makes the algorithm more prone to getting trapped in local optima, thereby compromising convergence accuracy. Consequently, this study opts for a population size of 40 in subsequent experimental settings to better leverage the algorithm’s performance.

**Figure 8:**
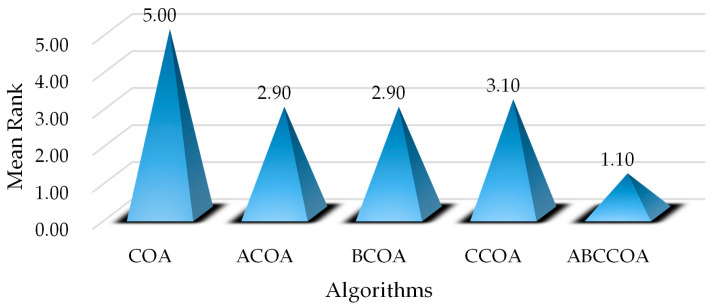
Average ranking of algorithms under different strategies.

Meanwhile, to validate the effectiveness of the proposed learning strategies, we define several modified algorithms based on the CO. Specifically, we introduce the adaptive search strategy into COA to form ACOA, incorporate the balancing factor to create BCOA, adopt the centroid guidance strategy to develop CCOA, and integrate all three learning strategies to establish ABCCOA. Subsequently, we conduct experiments on these defined algorithms using the CEC2020 benchmark suite. The results are illustrated in Figure 8, which clearly shows that introducing each individual learning strategy into COA enhances the algorithm’s performance, confirming the effectiveness of each strategy in improving algorithmic performance. Furthermore, it is noteworthy that when all three learning strategies are integrated simultaneously, the algorithm exhibits superior optimization performance compared to when only a single strategy is employed. The aforementioned discussion substantiates the effectiveness of each learning strategy proposed in this paper and demonstrates that integrating all three strategies can further enhance the algorithm’s performance.

### 4.2. Population Diversity Analysis

**Figure 9:**
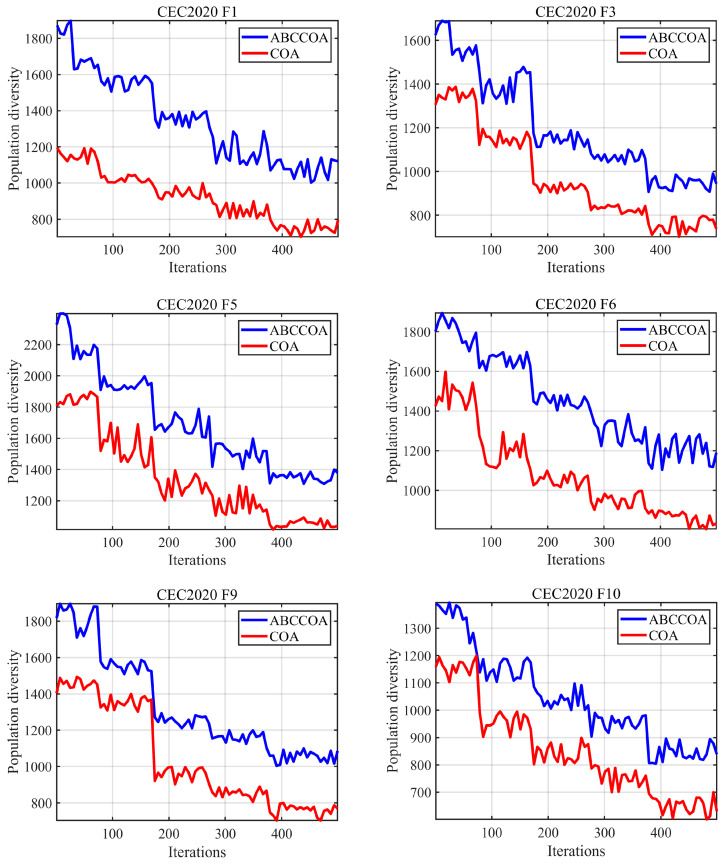
Comparison chart of population diversity.

In this section, we primarily analyze the population diversity of the ABCCOA algorithm during the execution of optimization problems. In the context of solving optimization problems, a higher population diversity indicates that the algorithm possesses a stronger global search capability, which helps it to escape local optima traps and thereby enhances the optimization precision of the algorithm. The experimental results are illustrated in Figure 9, where the x-axis represents the number of iterations, and the y-axis denotes the population diversity during the algorithm’s execution for the optimization problem. The blue line depicts the changes in population diversity for the ABCCOA algorithm, while the red line represents the same for the COA algorithm.

As depicted in the figure, during the early iterations of solving both unimodal and complex multimodal optimization problems, the ABCCOA algorithm exhibits higher population diversity compared to the COA algorithm. This is primarily attributed to the adaptive search strategy proposed in this study, which leverages its gap-learning mechanism to enhance the algorithm’s global search capabilities. This enables the ABCCOA algorithm to effectively locate potential optimal solution regions in the early iterations, thereby improving optimization precision. Subsequently, as shown in the figure, the ABCCOA algorithm maintains higher population diversity than the COA algorithm in the later iterations. This is advantageous for enhancing the algorithm’s ability to escape local optima traps. The introduction of the balancing factor in this study coordinates the exploration and exploitation phases, ensuring that the ABCCOA algorithm retains a certain degree of global search capability in the later iterations. This strengthens its ability to escape local optima and mitigates premature convergence. In summary, these observations confirm the effectiveness of the proposed adaptive search strategy and balancing factor in improving global exploration capabilities and enhancing the algorithm’s ability to escape local optima. They also validate that, compared to the COA algorithm, the ABCCOA algorithm achieves higher population diversity, leading to superior optimization performance.

### 4.3. Exploration/Exploitation Balance Analysis

**Figure 10:**
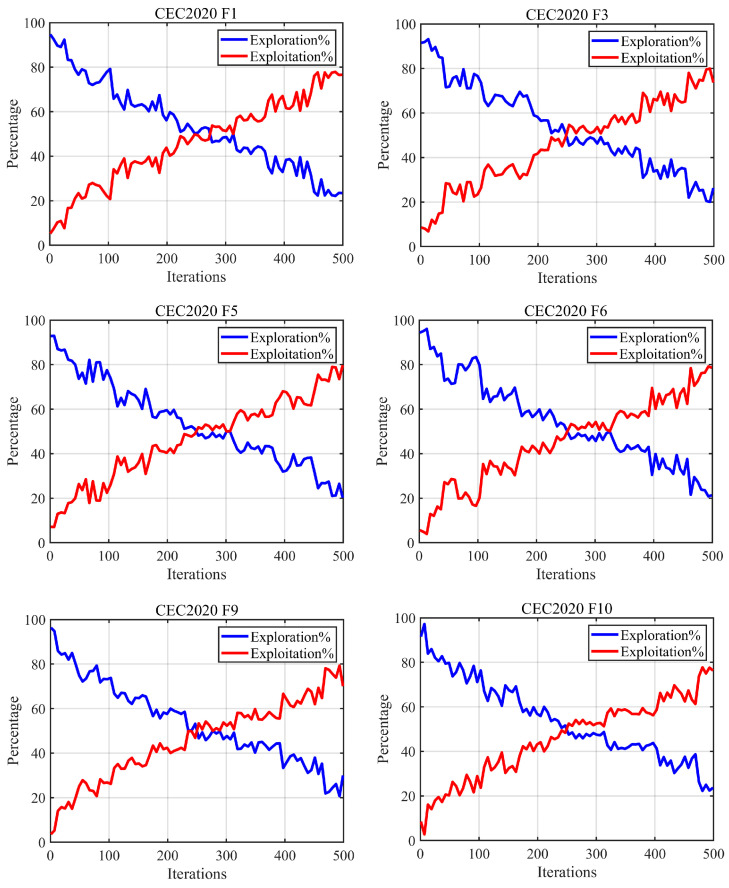
Exploration/exploitation ratio analysis plot.

In this section, we primarily analyze the balance between the exploration and exploitation phases of the ABCCOA algorithm during the execution of optimization problems. An effective optimization algorithm should typically maintain a well-balanced trade-off between exploration and exploitation. Specifically, in the early iterations, the algorithm should focus on global exploration to identify superior regions containing potential optimal solutions. In the later iterations, it should prioritize local exploitation to refine the regions identified during the exploration phase, thereby enhancing optimization precision and convergence speed. Simultaneously, the algorithm must retain a certain level of global search capability to ensure its ability to escape local optima traps. The experimental results are illustrated in Figure 10, where the x-axis represents the number of iterations, and the y-axis denotes the exploration-to-exploitation ratio. The blue line indicates the global exploration rate, while the red line represents the local exploitation rate.

As depicted in Figure 10, when solving unimodal and complex multimodal optimization problems, the ABCCOA algorithm exhibits a dominant global exploration phase in the early iterations. During this phase, the algorithm effectively identifies promising regions containing optimal solutions, primarily due to the enhanced global search capabilities conferred by the adaptive search strategy proposed in this study. As iterations progress, the global exploration rate gradually decreases while the local exploitation rate increases, with the local exploitation phase becoming dominant. This facilitates the algorithm’s refinement of the potential optimal solution regions, thereby improving optimization precision and convergence speed. This improvement is largely attributed to the centroid guidance strategy, which strengthens the algorithm’s local exploitation capabilities. Notably, even in the later iterations when local exploitation dominates, the global exploration rate remains approximately 30%. This is primarily due to the introduction of the balancing factor, which ensures a well-coordinated trade-off between the exploration and exploitation phases throughout the algorithm’s execution. Consequently, the algorithm retains a certain level of global exploration capability in the later iterations, enhancing its ability to escape local optima traps. In summary, these observations confirm that the integration of the adaptive search strategy, balancing factor, and centroid guidance strategy in the ABCCOA algorithm enables a superior balance between the exploration and exploitation phases. This effectively improves the algorithm’s optimization precision while accelerating convergence.

### 4.4. Fitness Function Values Analysis on CEC2020

In this section, we primarily analyze the fitness function values of the ABCCOA algorithm when solving the CEC2020 test suite to comprehensively evaluate its optimization performance. The evaluation metrics primarily include the mean and standard deviation derived from 30 independent experimental runs. The experimental results are presented in Table A1, where “Mean” denotes the average fitness function value obtained across the 30 independent experiments, and “Std” represents the standard deviation of these 30 runs. Additionally, “Mean Rank” indicates the average ranking of the algorithm based on the mean fitness function values, while “Final Rank” reflects the algorithm’s overall performance ranking determined from the “Mean Rank.”

**Figure 11:**
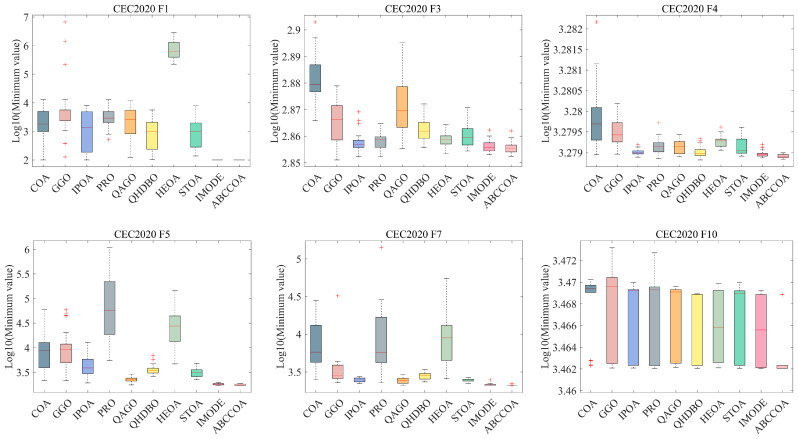
The boxplot on the CEC2020 test function.

**Figure 12:**
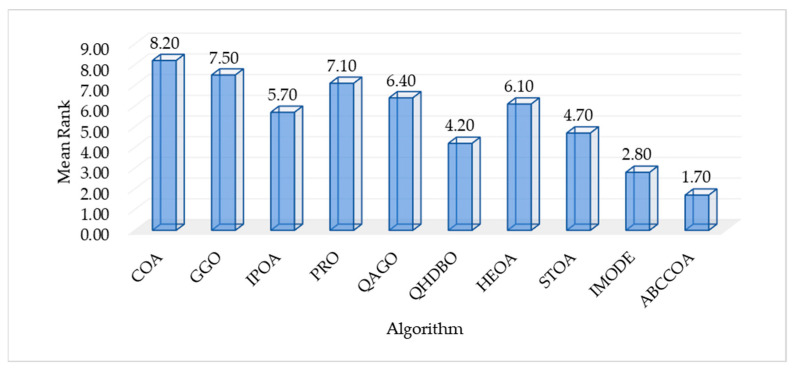
The mean rank on the CEC2020 test function.

As shown in the table, for the unimodal function F1, the ABCCOA algorithm achieves the optimal value compared to the benchmark algorithms, indicating that ABCCOA possesses stronger local exploitation capabilities. This is primarily attributed to the centroid guidance strategy proposed in this study, which effectively enhances the algorithm’s local exploitation performance, thereby improving optimization precision. Additionally, when solving complex multimodal functions F2 to F10, the ABCCOA algorithm ranks first in terms of the average fitness value across eight test functions compared to the benchmark algorithms, achieving a win rate of 88.8%. This success is largely due to the adaptive search strategy and balancing factor introduced in this study, which collectively improve the algorithm’s global search capabilities and its ability to escape local optima traps. This enables the algorithm to effectively escape modal traps and better locate regions containing potential optimal solutions. Meanwhile, Figure 11 visualizes the box plots of the algorithm’s results over 30 independent runs. As illustrated, in most cases, ABCCOA exhibits a lower box height and fewer outliers compared to the benchmark algorithms, confirming that the proposed ABCCOA algorithm demonstrates higher solution stability and concentration, ensuring its applicability in real-world optimization scenarios. Furthermore, to provide a more intuitive comparison of algorithm performance, Figure 12 visualizes the mean ranking plot. As shown, ABCCOA achieves a mean rank of 1.70, securing the first overall rank with the lowest bar height, outperforming the second-ranked IMODE by approximately 39.2%. In summary, these observations confirm that the adaptive search strategy, balancing factor, and centroid guidance strategy proposed in this study significantly enhance the algorithm’s performance. They also validate that the proposed ABCCOA is a robust optimization algorithm with efficient optimization capabilities, offering higher solution stability and broader applicability.

### 4.5. Nonparametric Test Analysis on CEC2020

**Figure 13:**
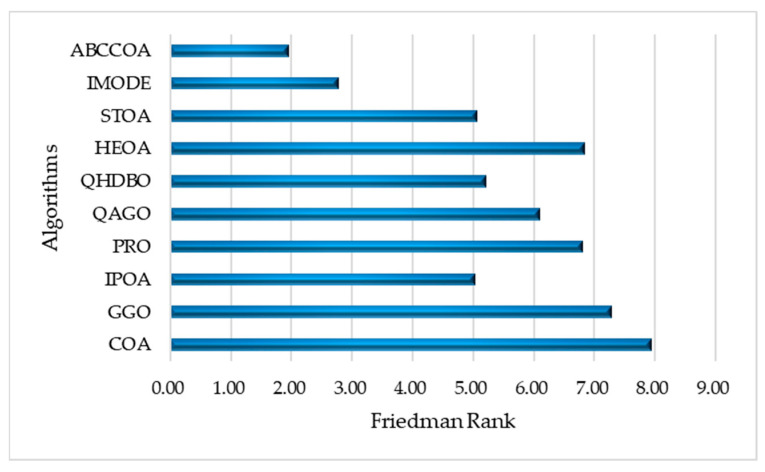
Friedman nonparametric test results on CEC2020 test function.

The optimization performance of the ABCCOA algorithm was primarily evaluated using numerical results in the preceding analysis. However, since outliers can influence numerical outcomes to some extent, this section employs nonparametric statistical tests to mitigate their impact on the algorithm’s numerical results. Specifically, we conduct Wilcoxon and Friedman nonparametric tests with a significance level of 0.05 on the results of 30 independent experimental runs to further assess the algorithm’s optimization performance. The results of the Wilcoxon nonparametric test are presented in Table A2, where “+” indicates that the performance of the benchmark algorithm is significantly superior to that of the proposed ABCCOA algorithm, “−” denotes that the benchmark algorithm’s performance is significantly inferior to ABCCOA, and “=” signifies no statistically significant difference in performance between the benchmark algorithm and ABCCOA. The outcomes of the Friedman nonparametric test are illustrated in Figure 13.

As shown in the table, the COA, GGO, and QAGO algorithms exhibit significantly inferior performance compared to the ABCCOA algorithm on nine test functions, representing a 90% ratio. The IPOA and PRO algorithms underperform ABCCOA on all ten test functions, achieving a 100% ratio. The QHDBO, HEOA, and STOA algorithms demonstrate weaker performance than ABCCOA on eight test functions, corresponding to an 80% ratio. In summary, the ABCCOA algorithm achieves superior optimization performance over the benchmark algorithms due to its advantageous learning strategies, positioning it as an efficient algorithm. However, it is noteworthy that the COA, GGO, QAGO, QHDBO, and HEOA algorithms outperform ABCCOA on the F8 test function, indicating that ABCCOA’s performance could be further enhanced for certain specific optimization problems. From a comprehensive perspective, across the 90 conducted tests, ABCCOA achieves victory in 78 cases, yielding an 86.6% success rate. This suggests that, despite the occasional shortcomings in specific optimization scenarios, ABCCOA is a robust algorithm with highly efficient optimization capabilities. Additionally, as illustrated by the Friedman nonparametric test results in Figure 13, ABCCOA attains a Friedman mean rank of 1.95, securing the top position. It outperforms the second-ranked IMODE algorithm by 29.8%, demonstrating superior optimization performance. The conclusions drawn from the nonparametric tests collectively validate that the adaptive search strategy, balancing factor, and centroid guidance strategy proposed in this study significantly enhance the algorithm’s performance. Furthermore, the ABCCOA algorithm can be regarded as an efficient optimization algorithm with robust performance.

### 4.6. Convergence Analysis on CEC2020

**Figure 14:**
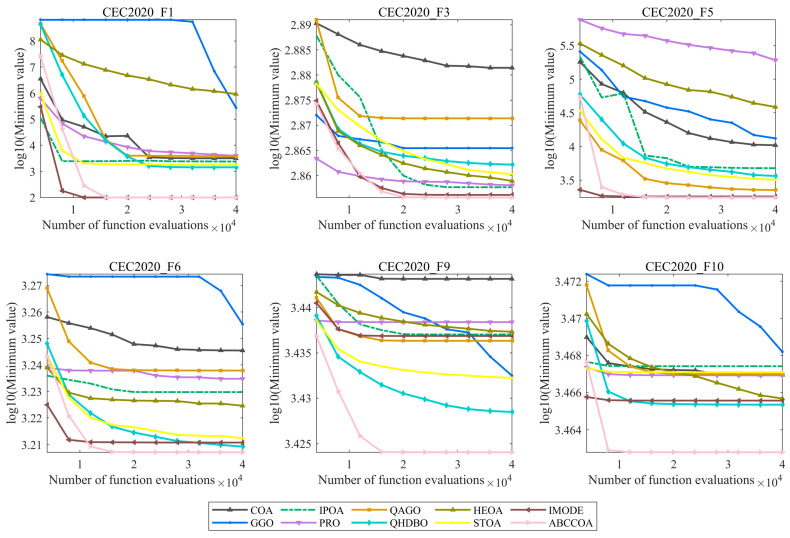
Convergence graph on CEC2020 test function.

The preceding sections primarily analyzed the ABCCOA algorithm’s population diversity, exploration/exploitation balance, fitness function values, solution stability, and nonparametric test results, collectively validating the algorithm’s efficient optimization capabilities. However, beyond these metrics, the convergence speed of the algorithm is also critical, as it reflects the algorithm’s practical applicability in real-world optimization scenarios. Therefore, this section focuses on analyzing the convergence behavior of the algorithm when solving the CEC2020 test function suite. The experimental results are depicted in Figure 14, where the x-axis represents the number of function evaluations, and the y-axis denotes the common logarithm (base 10) of the fitness function values.

As illustrated in the figure, all algorithms exhibit regional convergence behavior and effectively solve the optimization problems. However, it is noteworthy that after approximately 15,000 function evaluations, the ABCCOA algorithm demonstrates a distinct advantage, outperforming the benchmark algorithms in terms of convergence precision. This superiority is primarily attributed to the strategies proposed in this study, which enable the algorithm to rapidly locate regions containing potential optimal solutions, thereby enhancing convergence precision. Moreover, as iterations progress, the ABCCOA algorithm’s leading margin gradually widens, ultimately achieving stable and regionally consistent convergence behavior with a faster convergence rate. This is mainly due to the enhancement of the algorithm’s exploitation capabilities by the proposed strategies, which accelerates convergence. In summary, these observations confirm that the adaptive search strategy, balancing factor, and centroid guidance strategy proposed in this study significantly improve the algorithm’s convergence properties. Consequently, the ABCCOA algorithm can be regarded as an algorithm with favorable convergence characteristics and practical applicability.

The analyses conducted in the preceding sections—encompassing population diversity, exploration/exploitation balance, fitness function values, solution stability, nonparametric testing, and convergence behavior-validate the enhancing effects of the three proposed learning strategies on the ABCCOA algorithm. Furthermore, these analyses confirm that, compared to the benchmark algorithms, ABCCOA is a robust optimization tool characterized by efficient optimization performance, high solution stability, and rapid convergence properties.

### 4.7. Fitness Function Values Analysis on Real Problems

The optimization performance of the ABCCOA algorithm has already been validated on synthetic functions, as mentioned above. Additionally, to further assess the algorithm’s performance, this section evaluates the ABCCOA algorithm on five real-world engineering optimization problems, including the 10-bar truss design problem (RP1), Himmel Blau’s function (RP2), tension/compression spring design case 1 (RP3), tension/compression spring design case 2 (RP4), and the welded beam design problem (RP5). The experimental results are presented in Table A3. As can be seen from the table, the ABCCOA algorithm achieves optimal fitness function values across all five engineering optimization problems considered. Compared to the comparative algorithms, it demonstrates the highest win rate, further corroborating that the ABCCOA algorithm is an efficient optimizer with high-performance capabilities.

## 5. Experimental Results and Discussion on FS Problems

The preceding sections evaluated the optimization performance of the ABCCOA algorithm on the CEC2020 test function suite, confirming its efficacy as a high-performance optimization algorithm. In this section, the proposed ABCCOA algorithm is applied to solve FS problems. The primary objective is to minimize redundancy in the original dataset by identifying a compact subset of highly informative features, thereby enhancing classification accuracy while reducing computational costs. Specifically, the experiments were conducted on 27 FS datasets, and the performance of ABCCOA was compared against seven state-of-the-art algorithms. The details of the 27 FS datasets are provided in Table 3, and the benchmark algorithms are listed in Table 4. To ensure experimental fairness, the population size was set to 40, the maximum number of iterations was set to 100, and each experiment was independently repeated 30 times to aggregate the results. The FS performance of the ABCCOA algorithm was objectively evaluated by comprehensively considering the fitness function values, classification accuracy, feature subset size, and runtime metrics.

### 5.1. FS Problems Model

In this section, we focus on formulating the model for feature selection (FS) problems. FS plays a pivotal role in machine learning and data mining, as its primary objective is to reduce redundancy in the original dataset, thereby decreasing dataset complexity while enhancing classification accuracy. This process mitigates computational complexity in subsequent machine learning and data mining tasks and improves the generalization capability of model training. Specifically, the FS process involves searching through a vast space of feature subset combinations to identify a compact yet effective set of features that adequately represent the entire dataset, ultimately boosting model classification accuracy. To achieve this, we employ Equation (22) as the objective function for the FS problem.

(22) $$\min f(Y_{i})=\lambda_{1}\cdot error+\lambda_{2}\cdot R/n$$

where $Y_{i}$ denotes the *ith* FS subset combination, *error* represents the classification error rate when using $Y_{i}$, *R* indicates the size of the FS subset combination, *n* denotes the number of features in the original dataset, $\lambda_{1}$ is a constant within the interval [0, 1], and $\lambda_{2}=1-\lambda_{1}$. In this paper, $\lambda_{1}$ is set to 0.9.

**Figure 15:**
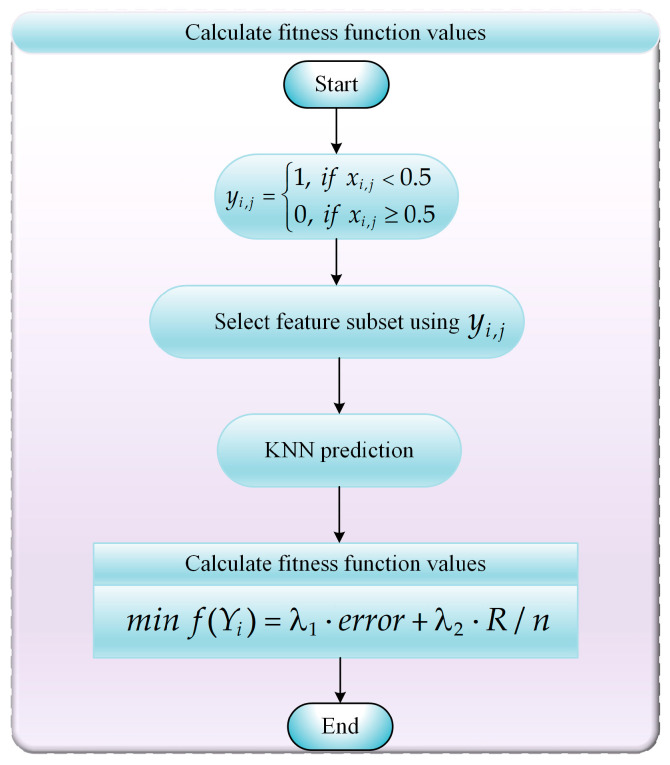
Calculation process of fitness function value for FS problem.

The FS problem is a classic combinatorial optimization task that involves selecting a subset of features from the original dataset and subsequently evaluating the quality of this subset by computing an objective function value using the selected features. In this section, we apply the proposed ABCCOA algorithm to solve the FS problem. Consequently, it is necessary to convert the real-valued solutions generated by the ABCCOA algorithm during iterations into binary (0–1) values, which indicate whether a feature is selected or not. Below, we detail the step-by-step procedure for selecting feature subsets and computing the fitness function values when solving the FS problem using the ABCCOA algorithm. The flowchart is illustrated as shown in Figure 15.

**Step 1**: Using Equation (23), we convert real-valued individual $X_{i}=(x_{i,1},x_{i,2},\ldots ,x_{i,j},\ldots ,x_{i,Dim})$ in the ABCCOA algorithm into a binary individual $Y_{i}=(y_{i,1},y_{i,2},\ldots ,y_{i,j},\ldots ,y_{i,Dim})$.
  (23) $$y_{i,j}=\left\{\begin{array}{l} 1\;if\;x_{i,j}<0.5\;\\ 0\;if\;x_{i,j}\geq 0.5\; \end{array}\right.,\;i=1,2,\ldots ,N,\;j=1,2,\ldots ,Dim.$$
**Step 2**: The binary individual $Y_{i}=(y_{i,1},y_{i,2},\ldots ,y_{i,j},\ldots ,y_{i,Dim})$ is utilized to select a feature subset combination from the original dataset. Here, $y_{i,j}=1$ indicates that the *jth* feature in the original dataset is selected in the *ith* feature subset combination, whereas $y_{i,j}=0$ indicates that the *jth* feature is not selected.
**Step 3**: The classification accuracy of the selected feature subset combinations is computed using a K-Nearest Neighbors (KNN) classifier, where *K* is set to 5.
**Step 4**: The objective function value for the feature subset combination is computed using Equation (22) with the information output by the KNN classifier.

### 5.2. Fitness Function Values Analysis of FS Problems

In this section, the focus is on analyzing the fitness function values of the ABCCOA algorithm when solving FS problems. Specifically, the algorithm was tested on 27 FS datasets encompassing low-dimensional, medium-dimensional, and high-dimensional cases while being compared with seven other algorithms known for their high efficiency. The experimental results are presented in Table A4. Here, “MIN,” “AVG,” and “MAX” denote the minimum, average, and maximum fitness function values obtained from 30 independent experimental runs, respectively. “Mean Rank” represents the average ranking of the algorithm across the 27 FS problem instances, and “Final Rank” indicates the algorithm’s ultimate ranking based on the “Mean Rank” metric.

As can be observed from the table, when solving low-dimensional FS problems, the ABCCOA algorithm ranks first on seven FS problems in terms of the optimal fitness function value, achieving a win rate of 87.5%. This is primarily attributed to the introduction of the centroid guidance strategy in this paper, which enhances the algorithm’s local exploitation capability, enabling it to explore more suitable combinations of feature subsets. Meanwhile, in terms of the average fitness function value, the ABCCOA algorithm ranks first on eight FS problems, with a ratio of 100%, indicating its high solution stability when solving FS problems. Additionally, in terms of the worst fitness function value, the ABCCOA algorithm ranks first on six FS problems, with a ratio of 75%, demonstrating its higher fault tolerance compared to the comparative algorithms when solving FS problems. In summary, it can be concluded that, due to the incorporation of the three learning strategies proposed in this paper, the ABCCOA algorithm is capable of obtaining excellent feature subset combinations for low-dimensional FS problems while showcasing its robust solution stability and fault tolerance.

When solving medium-dimensional FS problems, algorithms often require better global search capabilities to effectively explore superior potential optimal regions. As evident from the table, in terms of the optimal fitness function value for medium-dimensional FS problems, the ABCCOA algorithm ranks first on eight FS problems, accounting for 100%, demonstrating its stronger global optimization capability. Furthermore, in terms of the average fitness function value, the ABCCOA algorithm ranks first on seven FS problems, with a ratio of 87.5%, exhibiting greater solution stability that facilitates its implementation in practical application scenarios. In terms of the worst fitness function value, the ABCCOA algorithm ranks first on six FS problems, with a ratio of 75%, showcasing its stronger solution fault tolerance and robustness.

When addressing high-dimensional FS problems where the number of features exceeds 100, algorithms demand a higher balance between global exploration and local exploitation. Firstly, in terms of the optimal fitness function value, the ABCCOA algorithm ranks first on nine FS problems, with a ratio of 81.8%, demonstrating its stronger global optimization capability. This is largely attributed to the balance factor proposed in this paper, which achieves a good equilibrium between the exploration and local exploitation phases, thereby enhancing its search accuracy. Additionally, in terms of the average fitness function value, the ABCCOA algorithm ranks first on nine FS problems, with a ratio of 81.8%, achieving better overall FS performance compared to the comparative algorithms and exhibiting stronger solution stability. In terms of the worst fitness function value, the ABCCOA algorithm ranks first on eight FS problems, with a ratio of 72.7%, demonstrating its stronger solution fault tolerance and robustness compared to the comparative algorithms.

**Figure 16:**
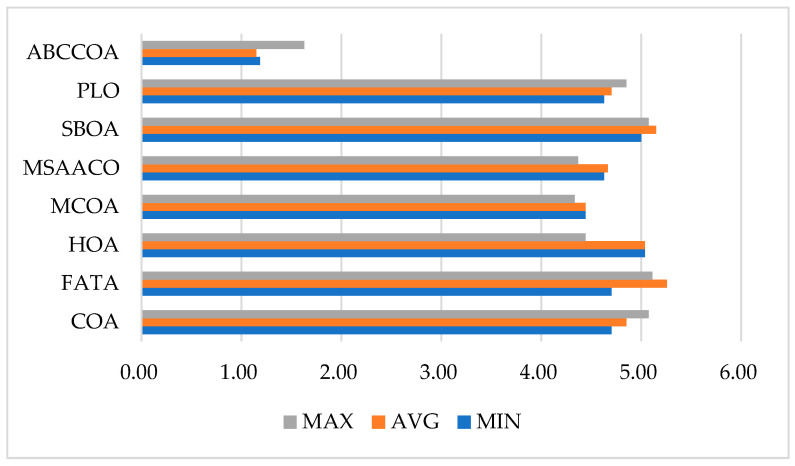
Ranking of fitness values on FS problems.

Meanwhile, Figure 16 visualizes the average rankings of the algorithms across different metrics. As can be seen from the figure, the ABCCOA algorithm achieves significant advantages in terms of the optimal, average, and worst fitness function values with the lowest bar heights. In conclusion, it can be concluded that the ABCCOA algorithm proposed in this paper is a robust and stable algorithm with efficient feature search performance, exhibiting higher solution stability and fault tolerance.

### 5.3. Classification Accuracy and Feature Subset Size Analysis of FS Problems

The analysis of the fitness function values of the ABCCOA algorithm when solving FS problems in the previous subsection has confirmed the algorithm’s high efficiency. The FS problem aims to extract the most representative feature subset to characterize the entire dataset, thereby improving classification accuracy. Therefore, in this section, we primarily evaluate the two most crucial metrics in FS problems: classification accuracy and the size of the feature subset. The experimental results for classification accuracy are presented in Table A5, while those for the size of the feature subset are shown in Table A6. Here, “Mean Rank” denotes the average ranking of the algorithm across 27 FS problems, and “Final Rank” represents the algorithm’s ultimate ranking based on the “Mean Rank” metric.

**Figure 17:**
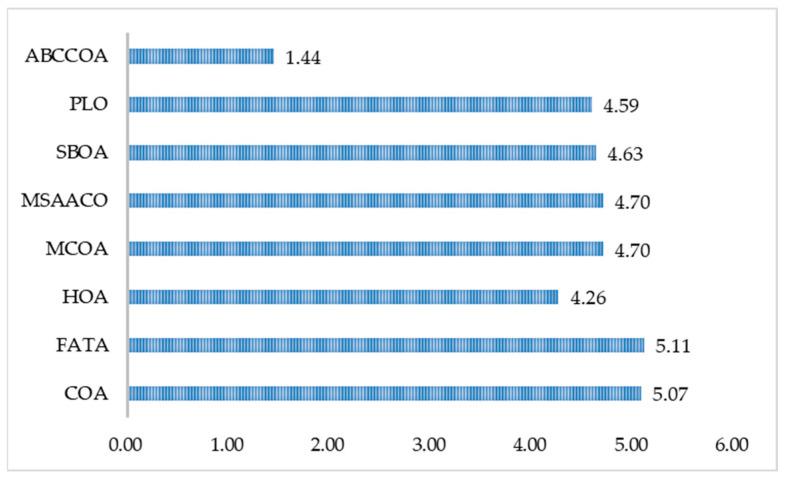
The average ranking of classification accuracy on FS problems.

**Figure 18:**
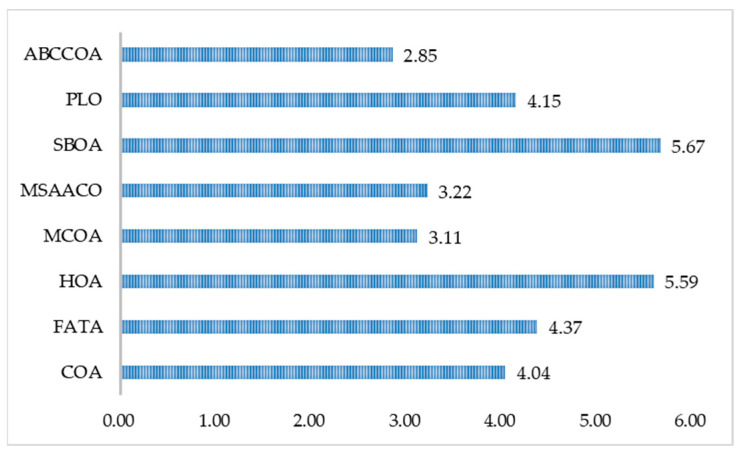
Average ranking of feature subset size on FS problem.

As can be observed from Table A5, when solving low-dimensional FS problems, the ABCCOA algorithm achieved the optimal classification accuracy on seven FS problems, accounting for 87.5%. Compared to the comparative algorithms, it obtained a more superior combination of feature subsets, thereby achieving higher classification accuracy with fewer features. This is primarily attributed to the strategies proposed in this paper, which effectively enhance the algorithm’s local exploitation capability. Meanwhile, as revealed in Table A6, all algorithms were capable of effectively reducing the size of the feature subsets. However, the ABCCOA algorithm did not have a distinct advantage in terms of feature reduction. This is mainly because other algorithms filtered out highly informative features, which, despite their advantage in feature subset size, resulted in a significant loss of classification accuracy. This also highlights the insufficient global trade-off capability of the comparative algorithms, leading to the deletion of useful feature information. The ABCCOA algorithm, due to its higher global search capability, was able to strike a reasonable balance between classification accuracy and feature subset size, thereby preserving effective features. When addressing medium-dimensional FS problems, the ABCCOA algorithm ranked first on four FS problems, accounting for 50%, demonstrating a relatively high advantage compared to the comparative algorithms. Nevertheless, it must be acknowledged that the ABCCOA algorithm has certain limitations on specific datasets, and its performance still needs improvement. However, from a comprehensive perspective, it exhibits higher overall performance compared to the comparative algorithms. This is largely attributed to the learning strategies proposed in this paper, which effectively enhance the algorithm’s global search performance. Furthermore, when solving high-dimensional FS problems, the ABCCOA algorithm ranked first in terms of accuracy on eight FS problems, accounting for 72.7%, achieving higher performance compared to the comparative algorithms and effectively preserving key features. Meanwhile, Figure 17 and Figure 18, respectively, illustrate the average rankings of the algorithms in terms of classification accuracy and feature subset size. It can be observed that the ABCCOA algorithm holds a significant advantage in terms of classification accuracy. This is primarily due to the strategies proposed in this paper, which improve the algorithm’s global optimization capability and, consequently, enhance its FS performance. Additionally, in terms of the feature subset size metric, the ABCCOA algorithm ranks first, effectively reducing the dimensionality of features. In summary, the ABCCOA algorithm is capable of effectively reducing the dimensionality of the features in datasets when solving FS problems, thereby improving the algorithm’s classification accuracy. It can be considered a promising FS method.

### 5.4. Runtime Analysis of FS Problems 

**Figure 19:**
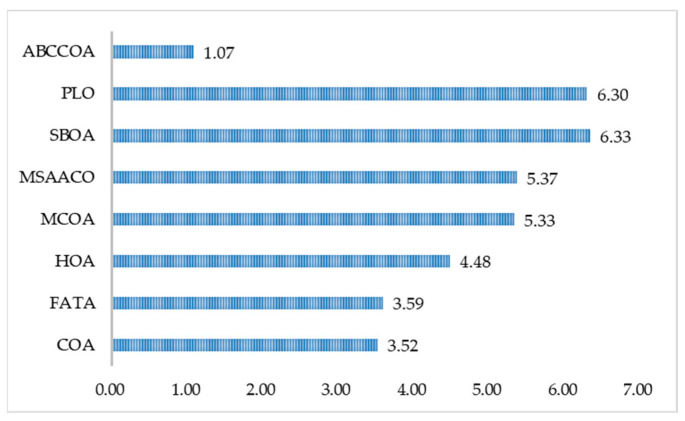
Average ranking of runtime on FS problem.

In this section, we primarily analyze the actual runtime of the ABCCOA algorithm when solving FS problems, which mainly reflects the algorithm’s real-time performance in practical applications. The experimental results are presented in Table A7, where “Mean Rank” denotes the average ranking of the algorithm in terms of runtime, and “Final Rank” represents the algorithm’s ultimate ranking based on the “Mean Rank” metric. As can be observed from the table, the ABCCOA algorithm achieved the shortest runtime on 25 FS problems, with a win rate of 92.5%. To more intuitively demonstrate the algorithm’s advantage in terms of runtime, Figure 19 displays a bar chart of the algorithms’ average rankings. From the figure, it is visually evident that the ABCCOA algorithm has a shorter bar length, indicating the shortest runtime. This confirms that the ABCCOA algorithm proposed in this paper is a real-time FS method.

### 5.5. Comprehensive Analysis of FS Problems

**Figure 20:**
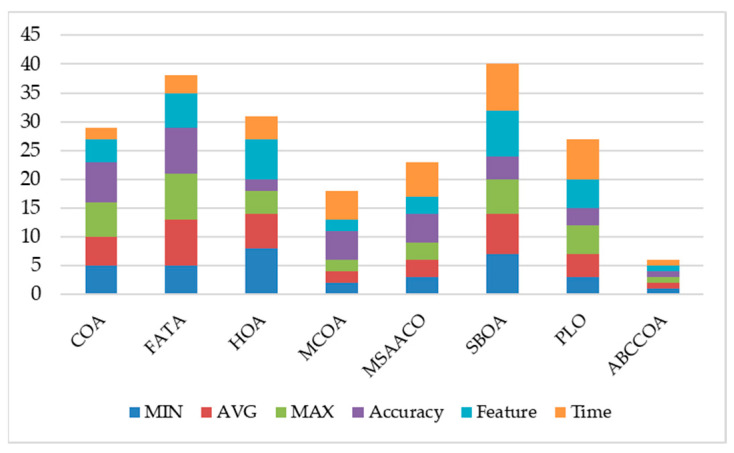
Comprehensive indicator analysis on FS problem.

In this section, a comprehensive analysis of the ABCCOA algorithm’s performance is conducted. By synthesizing the average rankings of the involved metrics, including the optimal fitness function value, average fitness function value, worst fitness function value, classification accuracy, feature subset size, and runtime, a stacked bar chart, as depicted in Figure 20, is formed. As can be observed from the figure, although the ABCCOA algorithm performs slightly worse than MCOA and MSAACO in terms of the feature subset size, its overall stacked bar exhibits the lowest height, indicating the best overall performance. This can be regarded as an FS algorithm with comprehensive and efficient performance.

## 6. Conclusions and Future Works

This paper addresses the limitations of the original COA in solving global optimization and FS problems, which stem from insufficient global exploration, inadequate local exploitation, and an imbalance between the exploration and exploitation phases. These issues often lead the algorithm to converge to suboptimal feature subset combinations, resulting in low classification accuracy and suboptimal optimization precision. To overcome these challenges, an enhanced COA algorithm, termed ABCCOA, is proposed by integrating three learning strategies. Firstly, to tackle the problem of inadequate global search performance in the COA algorithm when solving FS problems, which arises from the exponential increase in the number of feature subset combinations and consequently reduces classification accuracy, an adaptive search strategy is introduced. This strategy combines individual learning capabilities with the learnability of disparities, effectively enhancing the algorithm’s global exploration capabilities in solving FS problems and thereby improving the classification accuracy of feature subsets. Secondly, to address the issue of the COA algorithm’s tendency to converge to suboptimal feature subset combinations due to an imbalance between the exploration and exploitation phases when solving FS problems, a balancing factor is proposed for control. This factor’s phase is modulated using sine and cosine functions, while its dynamic adjustment is achieved through the integration of arcsine and arccosine functions. This approach ensures a well-balanced transition between the two phases, reducing the likelihood of the algorithm converging to suboptimal FS subsets. Lastly, to counter the problem of increased classification error rates resulting from insufficient local exploitation in the COA algorithm when solving FS problems, a centroid guidance strategy is introduced to enhance the algorithm’s local exploitation capabilities. By leveraging the centroids of individuals from the previous four generations and utilizing fractional-order historical memory, this strategy not only reduces the classification error rate of FS subsets but also accelerates convergence speed. Subsequently, the ABCCOA algorithm is applied to the CEC2020 test functions and five engineering optimization problems, demonstrating an optimization win rate exceeding 90% and a faster convergence speed compared to comparative algorithms, thereby confirming its efficient optimization performance. Furthermore, the ABCCOA algorithm, with its proven efficient optimization capabilities, was employed to solve 27 FS problems to evaluate its FS performance. The experimental results indicate that, compared to comparative algorithms, the ABCCOA algorithm exhibits significant advantages in terms of the optimal fitness function value, average fitness function value, worst fitness function value, classification accuracy, feature subset size, and runtime, achieving remarkable FS performance. Consequently, ABCCOA can be regarded as an efficient and robust FS algorithm.

However, despite ABCCOA achieving the best overall performance in solving FS problems, there is still room for improvement in its performance on certain specific FS datasets. Meanwhile, although the algorithm proposed in this paper may still offer advantages in combinatorial optimization processes within forward-looking fields, no further extended testing has been conducted in this regard. Therefore, the future work outlined in this paper primarily focuses on the following three aspects: 1. Developing strategies with more efficient search capabilities for specific FS problems to enhance their classification accuracy. 2. Extending the application of the ABCCOA algorithm to a broader range of combinatorial optimization problems, thereby expanding the application scenarios of the algorithm proposed in this paper. Examples include airline scheduling and photovoltaic parameter identification. 3. This paper has only developed a single-objective optimization model for the algorithm. In subsequent work, we will also extend it to a multi-objective model to further enhance the algorithm’s performance.

## Figures and Tables

**Table 1:** CEC2020 test function-specific information.

Functions	Type	Name	Values
CEC2020_F1	Unimodal	Shifted and Rotated Bent Cigar Function	100
CEC2020_F2	Multi-modal	Shifted and Rotated Schwefel’s Function	1100
CEC2020_F3		Shifted and Rotated unacek bi-Rastrigin Function	700
CEC2020_F4		Expanded Rosenbrock’s plus Griewangk’s Function	1900
CEC2020_F5	Hybrid	Hybrid Function 1 (*N* = 3)	1700
CEC2020_F6		Hybrid Function 2 (*N* = 4)	1600
CEC2020_F7		Hybrid Function 3 (*N* = 5)	2100
CEC2020_F8	Composition	Composition Function 1 (*N* = 3)	2200
CEC2020_F9		Composition Function 2 (*N* = 4)	2400
CEC2020_F10		Composition Function 3 (*N* = 5)	2500

**Table 2:** Comparison of algorithm parameter settings on cec2020 test functions.

Algorithms	Time	Parameters Setting
COA [63]	2023	No Parameters
GGO [65]	2024	α=0.99, β=0.01
HEOA [66]	2024	$\alpha =4,\;\gamma =1.5,\;\omega =0.2\cos\left(\frac{\pi }{2}\left(1-\frac{t}{Max_{iter}}\right)\right)$
IPOA [67]	2024	$I\in \{1,2\},\;F_{PK}=0.4,\;F_{GK}=0.6$
PRO [68]	2024	$\tau =\frac{FEs}{MaxFEs},\;SR=e^{-(1-\tau )}$
QAGO [69]	2024	$P_{1}=\left\lfloor rand(0.05,0.2)\cdot N\right\rfloor ,\;P_{2}=Gaussian(0.001,0.001),\;P_{2}=Gaussian(rand(0,0.3),0.01)$
QHDBO [70]	2024	$R=(\cos(\pi \cdot (t/T_{max}))+1)\cdot 0.5$
STOA [71]	2019	$C_{f}=2,\;R_{rnd}\in [0,1],\;u=1,\;\upsilon =1$
IMODE [72]	2020	D=2, arch_rate=2.6

**Table 3:** Detailed information on 27 FS datasets.

Type	Name	Feature Number	Classification Number	Instance Size
Low	Aggregation	2	7	788
	Banana	2	2	5300
	Iris	4	3	150
	Bupa	6	2	345
	Glass	9	7	214
	Breastcancer	9	2	699
	Lipid	10	2	583
	HeartEW	13	2	270
Medium	Zoo	16	7	101
	Vote	16	2	435
	Congress	16	2	435
	Lymphography	18	4	148
	Vehicle	18	4	846
	WDBC	30	2	569
	BreastEW	30	2	569
	SonarEW	60	2	208
High	Libras	90	15	360
	Hillvalley	100	2	606
	Musk	166	2	476
	Clean	167	2	476
	Semeion	256	10	1593
	Madelon	500	2	2600
	Isolet	617	26	1559
	Lung	12,533	5	203
	MLL	12,582	3	72
	Ovarian	15154	2	253
	CNS	7129	2	60

**Table 4:** Comparison of algorithm parameter setting information on FS problems.

Algorithms	Time	Parameters Setting
COA [63]	2023	No Parameters
FATA [73]	2024	No Parameters
HOA [74]	2024	No Parameters
MCOA [75]	2024	$C_{2}=2-(FEs/MaxFEs),\;C_{3}=3,\;k=(1+(FEs/MaxFEs{)}^{0.5}{)}^{10}$
MSAACO [76]	2024	α=1, β=7
SBOA [77]	2024	RL=0.5·Levy(Dim), K∈{1,2}
PLO [78]	2024	$W_{1}=\frac{2}{\left(1+e^{-2{\left(t/T\right)}^{4}}\right)}-1,\;W_{2}=e^{-{\left(2t/T\right)}^{3}}$

## Data Availability

All experimental data can be obtained by contacting the corresponding author.

## Appendix A

**Table A1:** The fitness function value on the CEC2020 test function.

Problems	Metrics	COA	GGO	IPOA	PRO	QAGO	QHDBO	HEOA	STOA	IMODE	ABCCOA
CEC2020_F1	Mean	3.1909 × 10^3^	2.8022 × 10^5^	2.3920 × 10^3^	4.0006 × 10^3^	3.7112 × 10^3^	1.4334 × 10^3^	9.2230 × 10^5^	1.7426 × 10^3^	1.0000 × 10^2^	1.0000 × 10^2^
	Std	3.3719 × 10^3^	1.2304 × 10^6^	2.4564 × 10^3^	3.0737 × 10^3^	3.5303 × 10^3^	1.5126 × 10^3^	6.8025 × 10^5^	2.1007 × 10^3^	0.0000 × 10^0^	1.0556 × 10^−14^
CEC2020_F2	Mean	1.9082 × 10^3^	1.8364 × 10^3^	1.4853 × 10^3^	1.4333 × 10^3^	1.9506 × 10^3^	1.6920 × 10^3^	1.2850 × 10^3^	1.8119 × 10^3^	1.3453 × 10^3^	1.2784 × 10^3^
	Std	3.3197 × 10^2^	3.3372 × 10^2^	2.1359 × 10^2^	1.8320 × 10^2^	2.6218 × 10^2^	1.6673 × 10^2^	1.0412 × 10^2^	2.6013 × 10^2^	2.1524 × 10^2^	1.1347 × 10^2^
CEC2020_F3	Mean	7.6108 × 10^2^	7.3357 × 10^2^	7.2055 × 10^2^	7.2118 × 10^2^	7.4366 × 10^2^	7.2806 × 10^2^	7.2263 × 10^2^	7.2482 × 10^2^	7.1801 × 10^2^	7.1718 × 10^2^
	Std	1.5046 × 10^1^	1.2631 × 10^1^	6.1067 × 10^0^	4.3600 × 10^0^	1.8048 × 10^1^	6.4219 × 10^0^	4.5734 × 10^0^	6.8581 × 10^0^	3.8629 × 10^0^	3.5531 × 10^0^
CEC2020_F4	Mean	1.9045 × 10^3^	1.9033 × 10^3^	1.9011 × 10^3^	1.9017 × 10^3^	1.9017 × 10^3^	1.9011 × 10^3^	1.9022 × 10^3^	1.9016 × 10^3^	1.9009 × 10^3^	1.9007 × 10^3^
	Std	2.9976 × 10^0^	1.5294 × 10^0^	3.1868 × 10^−1^	8.0634 × 10^−1^	6.9471 × 10^−1^	5.5801 × 10^−1^	5.4287 × 10^−1^	8.4758 × 10^−1^	3.4417 × 10^−1^	2.1239 × 10^−1^
CEC2020_F5	Mean	1.0452 × 10^4^	1.3219 × 10^4^	4.7648 × 10^3^	1.9134 × 10^5^	2.2577 × 10^3^	3.6506 × 10^3^	3.8663 × 10^4^	3.1948 × 10^3^	1.8119 × 10^3^	1.7500 × 10^3^
	Std	1.0876 × 10^4^	1.5027 × 10^4^	2.6024 × 10^3^	2.7411 × 10^5^	2.5618 × 10^2^	9.3224 × 10^2^	3.6543 × 10^4^	6.6865 × 10^2^	6.4939 × 10^1^	5.4569 × 10^1^
CEC2020_F6	Mean	1.7598 × 10^3^	1.8005 × 10^3^	1.6973 × 10^3^	1.7170 × 10^3^	1.7293 × 10^3^	1.6193 × 10^3^	1.6773 × 10^3^	1.6311 × 10^3^	1.6249 × 10^3^	1.6113 × 10^3^
	Std	1.0650 × 10^2^	1.1254 × 10^2^	6.9794 × 10^1^	7.4794 × 10^1^	1.0833 × 10^2^	3.1798 × 10^1^	7.9426 × 10^1^	4.9920 × 10^1^	4.7920 × 10^1^	2.9837 × 10^1^
CEC2020_F7	Mean	9.1304 × 10^3^	5.0144 × 10^3^	2.4814 × 10^3^	1.4209 × 10^4^	2.4609 × 10^3^	2.8087 × 10^3^	1.1044 × 10^4^	2.4544 × 10^3^	2.1410 × 10^3^	2.1062 × 10^3^
	Std	7.1463 × 10^3^	7.4174 × 10^3^	1.7018 × 10^2^	2.5252 × 10^4^	2.3036 × 10^2^	2.8361 × 10^2^	1.0598 × 10^4^	1.1367 × 10^2^	8.0007 × 10^1^	2.1880 × 10^1^
CEC2020_F8	Mean	2.3080 × 10^3^	2.3057 × 10^3^	2.3100 × 10^3^	2.3100 × 10^3^	2.3072 × 10^3^	2.3077 × 10^3^	2.3030 × 10^3^	2.3068 × 10^3^	2.3069 × 10^3^	2.3100 × 10^3^
	Std	1.0866 × 10^1^	1.8825 × 10^1^	1.0581 × 10^−12^	2.3739 × 10^−3^	1.5463 × 10^1^	1.2572 × 10^1^	2.3518 × 10^1^	1.7244 × 10^1^	1.7146 × 10^1^	4.5475 × 10^−13^
CEC2020_F9	Mean	2.7744 × 10^3^	2.7070 × 10^3^	2.7354 × 10^3^	2.7440 × 10^3^	2.7310 × 10^3^	2.6821 × 10^3^	2.7374 × 10^3^	2.7055 × 10^3^	2.7344 × 10^3^	2.6550 × 10^3^
	Std	1.9217 × 10^1^	1.0097 × 10^2^	4.4992 × 10^1^	4.7359 × 10^1^	7.9166 × 10^1^	9.2976 × 10^1^	5.8952 × 10^1^	7.8665 × 10^1^	4.4548 × 10^1^	1.1648 × 10^2^
CEC2020_F10	Mean	2.9306 × 10^3^	2.9390 × 10^3^	2.9337 × 10^3^	2.9304 × 10^3^	2.9309 × 10^3^	2.9197 × 10^3^	2.9220 × 10^3^	2.9315 × 10^3^	2.9212 × 10^3^	2.9026 × 10^3^
	Std	4.7628 × 10^1^	2.6650 × 10^1^	2.1468 × 10^1^	2.4738 × 10^1^	2.1773 × 10^1^	2.2733 × 10^1^	2.0299 × 10^1^	2.1700 × 10^1^	2.3416 × 10^1^	1.3847 × 10^1^
Mean Rank		8.20	7.50	5.70	7.10	6.40	4.20	6.10	4.70	2.80	1.70
Final Rank		10	9	5	8	7	3	6	4	2	1

**Table A2:** Wilcoxon nonparametric test results on CEC2020 test function.

Problems	COA	GGO	IPOA	PRO	QAGO	QHDBO	HEOA	STOA	IMODE
CEC2020_F1	−	−	−	−	−	−	−	−	=
CEC2020_F2	−	−	−	−	−	−	=	−	−
CEC2020_F3	−	−	−	−	−	−	−	−	−
CEC2020_F4	−	−	−	−	−	−	−	−	−
CEC2020_F5	−	−	−	−	−	−	−	−	−
CEC2020_F6	−	−	−	−	−	−	−	−	=
CEC2020_F7	−	−	−	−	−	−	−	−	−
CEC2020_F8	+	+	−	−	+	+	+	+	=
CEC2020_F9	−	−	−	−	−	=	−	=	−
CEC2020_F10	−	−	−	−	−	−	−	−	−
+/−/=	1/9/0	1/9/0	0/10/0	0/10/0	1/9/0	1/8/1	1/8/1	1/8/1	0/7/3

**Table A3:** The fitness function value in real-world optimization problems.

Problems	Metrics	COA	GGO	IPOA	PRO	QAGO	QHDBO	HEOA	STOA	IMODE	ABCCOA
RP1	Mean	5.261 × 10^2^	5.347 × 10^2^	5.257 × 10^2^	5.599 × 10^2^	5.262 × 10^2^	5.247 × 10^2^	5.249 × 10^2^	5.360 × 10^2^	5.246 × 10^2^	5.245 × 10^2^
	Std	1.981 × 10^0^	8.537 × 10^0^	1.596 × 10^0^	1.022 × 10^1^	2.812 × 10^0^	1.118 × 10^0^	1.179 × 10^0^	6.981 × 10^0^	6.270 × 10^−02^	1.919 × 10^−2^
RP2	Mean	−3.067 × 10^4^	−3.067 × 10^4^	−3.067 × 10^4^	−3.005 × 10^4^	−3.067 × 10^4^	−3.067 × 10^4^	−3.067 × 10^4^	−3.067 × 10^4^	−3.067 × 10^4^	−3.067 × 10^4^
	Std	7.768 × 10^−7^	3.223 × 10^−4^	3.374 × 10^−11^	2.249 × 10^2^	1.100 × 10^−11^	1.110 × 10^−11^	1.110 × 10^−11^	3.598 × 10^−2^	1.042 × 10^−6^	1.392 × 10^−6^
RP3	Mean	1.280 × 10^−2^	1.274 × 10^−2^	1.267 × 10^−2^	2.226 × 10^−2^	1.268 × 10^−2^	1.267 × 10^−2^	1.267 × 10^−2^	1.314 × 10^−2^	1.267 × 10^−2^	1.267 × 10^−2^
	Std	1.402 × 10^−4^	1.021 × 10^−4^	5.570 × 10^−6^	8.296 × 10^−3^	2.380 × 10^−5^	5.254 × 10^−6^	5.682 × 10^−8^	4.130 × 10^−4^	1.170 × 10^−7^	4.378 × 10^−13^
RP4	Mean	2.691 × 10^0^	2.916 × 10^0^	2.664 × 10^0^	3.429 × 10^0^	2.660 × 10^0^	2.673 × 10^0^	2.668 × 10^0^	2.735 × 10^0^	2.660 × 10^0^	2.659 × 10^0^
	Std	5.083 × 10^−2^	3.480 × 10^−1^	1.415 × 10^−2^	4.178 × 10^−1^	7.474 × 10^−3^	5.196 × 10^−2^	1.761 × 10^−2^	9.737 × 10^−2^	7.470 × 10^−3^	1.712 × 10^−6^
RP5	Mean	1.670 × 10^0^	1.685 × 10^0^	1.670 × 10^0^	1.974 × 10^0^	1.670 × 10^0^	1.670 × 10^0^	1.670 × 10^0^	1.737 × 10^0^	1.670 × 10^0^	1.670 × 10^0^
	Std	2.497 × 10^−4^	6.173 × 10^−2^	1.187 × 10^−8^	1.204 × 10^−1^	5.800 × 10^−10^	2.258 × 10^−16^	2.182 × 10^−16^	4.694 × 10^−2^	1.950 × 10^−7^	1.934 × 10^−16^
Mean Rank		5.80	6.60	3.20	10.00	3.40	3.00	2.40	7.20	1.60	1.00
Final Rank		7	8	5	10	6	4	3	9	2	1

**Table A4:** The fitness function value on FS problem.

Datasets	Metrics	COA	FATA	HOA	MCOA	MSAACO	SBOA	PLO	ABCCOA
Aggregation	MIN	0.100	0.100	0.100	0.100	0.100	0.106	0.106	0.100
	AVG	0.100	0.100	0.100	0.100	0.100	0.106	0.106	0.100
	MAX	0.100	0.100	0.100	0.100	0.100	0.106	0.106	0.100
Banana	MIN	0.211	0.196	0.192	0.199	0.212	0.195	0.197	0.188
	AVG	0.211	0.196	0.192	0.199	0.212	0.195	0.197	0.188
	MAX	0.211	0.196	0.192	0.199	0.212	0.195	0.197	0.188
Iris	MIN	0.025	0.080	0.025	0.050	0.050	0.075	0.115	0.025
	AVG	0.025	0.088	0.025	0.050	0.051	0.075	0.115	0.025
	MAX	0.025	0.110	0.025	0.050	0.055	0.075	0.115	0.025
Bupa	MIN	0.285	0.285	0.333	0.333	0.307	0.350	0.311	0.285
	AVG	0.296	0.292	0.334	0.333	0.313	0.350	0.311	0.285
	MAX	0.344	0.311	0.337	0.333	0.337	0.350	0.311	0.285
Glass	MIN	0.312	0.290	0.269	0.259	0.259	0.312	0.248	0.226
	AVG	0.314	0.308	0.269	0.265	0.260	0.323	0.255	0.250
	MAX	0.333	0.356	0.269	0.280	0.270	0.344	0.312	0.270
Breastcancer	MIN	0.059	0.059	0.053	0.053	0.055	0.053	0.053	0.048
	AVG	0.060	0.060	0.063	0.053	0.055	0.053	0.053	0.050
	MAX	0.066	0.066	0.068	0.053	0.055	0.053	0.055	0.059
Lipid	MIN	0.245	0.247	0.253	0.229	0.245	0.237	0.258	0.222
	AVG	0.247	0.263	0.253	0.240	0.249	0.252	0.262	0.222
	MAX	0.251	0.268	0.257	0.261	0.265	0.263	0.268	0.222
HeartEW	MIN	0.090	0.140	0.190	0.147	0.138	0.113	0.122	0.105
	AVG	0.110	0.165	0.248	0.164	0.142	0.123	0.129	0.107
	MAX	0.147	0.190	0.281	0.188	0.147	0.154	0.156	0.122
Zoo	MIN	0.089	0.070	0.038	0.070	0.076	0.038	0.044	0.031
	AVG	0.121	0.095	0.041	0.073	0.114	0.071	0.065	0.035
	MAX	0.134	0.109	0.050	0.076	0.134	0.083	0.115	0.044
Vote	MIN	0.029	0.029	0.031	0.035	0.046	0.048	0.050	0.027
	AVG	0.042	0.049	0.035	0.045	0.050	0.061	0.054	0.027
	MAX	0.048	0.060	0.050	0.070	0.058	0.098	0.062	0.027
Congress	MIN	0.017	0.039	0.037	0.027	0.060	0.048	0.042	0.017
	AVG	0.017	0.052	0.038	0.027	0.065	0.048	0.048	0.025
	MAX	0.017	0.062	0.042	0.027	0.068	0.048	0.056	0.035
Lymphography	MIN	0.064	0.110	0.059	0.084	0.107	0.090	0.084	0.059
	AVG	0.086	0.132	0.076	0.090	0.139	0.113	0.103	0.067
	MAX	0.110	0.163	0.107	0.095	0.172	0.132	0.126	0.084
Vehicle	MIN	0.257	0.262	0.241	0.267	0.257	0.236	0.247	0.236
	AVG	0.275	0.285	0.266	0.279	0.273	0.251	0.257	0.251
	MAX	0.288	0.300	0.289	0.294	0.295	0.263	0.267	0.263
WDBC	MIN	0.061	0.037	0.069	0.066	0.046	0.068	0.056	0.037
	AVG	0.066	0.053	0.086	0.067	0.049	0.089	0.070	0.046
	MAX	0.075	0.064	0.102	0.070	0.058	0.103	0.081	0.055
BreastEW	MIN	0.039	0.036	0.037	0.035	0.039	0.041	0.039	0.021
	AVG	0.058	0.045	0.043	0.039	0.047	0.050	0.054	0.038
	MAX	0.067	0.051	0.055	0.056	0.057	0.061	0.064	0.059
SonarEW	MIN	0.054	0.125	0.057	0.049	0.034	0.035	0.044	0.015
	AVG	0.073	0.152	0.074	0.077	0.053	0.050	0.073	0.038
	MAX	0.093	0.189	0.089	0.091	0.081	0.062	0.099	0.050
Libras	MIN	0.153	0.138	0.238	0.204	0.178	0.271	0.164	0.092
	AVG	0.170	0.158	0.245	0.228	0.200	0.296	0.192	0.129
	MAX	0.194	0.178	0.259	0.262	0.218	0.328	0.224	0.144
Hillvalley	MIN	0.341	0.313	0.376	0.261	0.318	0.294	0.374	0.266
	AVG	0.360	0.323	0.391	0.281	0.331	0.310	0.396	0.298
	MAX	0.374	0.337	0.404	0.304	0.345	0.322	0.416	0.323
Musk	MIN	0.063	0.109	0.076	0.020	0.018	0.088	0.070	0.064
	AVG	0.093	0.133	0.098	0.033	0.028	0.109	0.090	0.080
	MAX	0.112	0.145	0.112	0.040	0.045	0.127	0.109	0.088
Clean	MIN	0.067	0.073	0.084	0.075	0.020	0.047	0.075	0.017
	AVG	0.089	0.091	0.094	0.103	0.044	0.078	0.083	0.027
	MAX	0.126	0.105	0.100	0.128	0.066	0.102	0.094	0.034
Semeion	MIN	0.114	0.106	0.131	0.070	0.119	0.089	0.080	0.063
	AVG	0.123	0.115	0.139	0.082	0.128	0.097	0.088	0.080
	MAX	0.133	0.123	0.145	0.096	0.135	0.110	0.093	0.106
Madelon	MIN	0.244	0.230	0.237	0.158	0.107	0.190	0.183	0.091
	AVG	0.268	0.262	0.251	0.201	0.121	0.209	0.204	0.109
	MAX	0.287	0.281	0.259	0.259	0.136	0.225	0.243	0.125
Isolet	MIN	0.161	0.151	0.178	0.164	0.065	0.136	0.134	0.060
	AVG	0.172	0.162	0.184	0.176	0.086	0.147	0.153	0.075
	MAX	0.184	0.177	0.189	0.184	0.110	0.157	0.178	0.097
Lung	MIN	0.072	0.051	0.051	0.115	0.087	0.047	0.038	0.021
	AVG	0.093	0.072	0.081	0.122	0.095	0.082	0.071	0.042
	MAX	0.124	0.093	0.098	0.135	0.113	0.120	0.113	0.063
MLL	MIN	0.081	0.071	0.082	0.072	0.101	0.123	0.053	0.041
	AVG	0.090	0.100	0.101	0.096	0.125	0.147	0.074	0.061
	MAX	0.101	0.117	0.125	0.123	0.145	0.160	0.092	0.082
Ovarian	MIN	0.110	0.061	0.101	0.071	0.062	0.041	0.051	0.012
	AVG	0.123	0.093	0.118	0.095	0.083	0.072	0.079	0.020
	MAX	0.180	0.118	0.123	0.114	0.098	0.081	0.100	0.034
CNS	MIN	0.118	0.084	0.071	0.088	0.080	0.117	0.101	0.061
	AVG	0.133	0.108	0.099	0.115	0.109	0.136	0.119	0.086
	MAX	0.153	0.126	0.119	0.142	0.137	0.150	0.125	0.091
Mean Rank	MIN	4.70	4.70	5.04	4.44	4.63	5.00	4.63	1.19
	AVG	4.85	5.26	5.04	4.44	4.67	5.15	4.70	1.15
	MAX	5.07	5.11	4.44	4.33	4.37	5.07	4.85	1.63
Final Rank	MIN	5	5	8	2	3	7	3	1
	AVG	5	8	6	2	3	7	4	1
	MAX	6	8	4	2	3	6	5	1

**Table A5:** Classification accuracy on FS problems.

Datasets	COA	FATA	HOA	MCOA	MSAACO	SBOA	PLO	ABCCOA
Aggregation	100.00	100.00	100.00	100.00	100.00	99.36	99.36	100.00
Banana	87.64	89.34	89.81	88.96	87.55	89.43	89.25	90.19
Iris	100.00	96.33	100.00	100.00	99.67	100.00	90.00	100.00
Bupa	73.04	72.90	66.81	66.67	69.28	66.67	71.01	73.04
Glass	69.05	69.52	73.81	75.00	76.19	68.57	75.00	76.19
Breastcancer	97.12	96.76	96.40	97.84	96.40	97.84	97.55	97.12
Lipid	74.31	73.62	74.31	75.52	75.00	74.66	72.84	77.59
HeartEW	90.56	85.37	74.63	85.19	87.41	90.37	88.89	92.41
Zoo	90.50	93.50	100.00	95.00	91.00	95.50	97.00	100.00
Vote	96.67	97.36	99.08	96.55	96.78	96.67	96.32	97.70
Congress	98.85	96.78	97.13	97.70	94.02	95.40	98.05	98.85
Lymphography	94.14	88.97	95.52	93.10	87.93	91.38	91.72	95.86
Vehicle	73.49	71.95	74.32	72.49	73.49	76.45	75.56	75.98
WDBC	94.07	96.55	92.48	93.63	95.75	92.21	93.54	96.64
BreastEW	96.37	98.23	99.29	98.05	96.99	97.96	96.99	99.20
SonarEW	95.61	85.37	98.29	93.90	96.10	98.54	95.12	98.05
Libras	83.75	84.72	79.31	76.53	79.58	71.39	82.64	87.78
Hillvalley	61.82	65.37	62.73	70.50	65.37	70.33	60.50	67.85
Musk	92.11	87.79	93.47	98.32	99.16	92.74	94.95	98.32
Clean	91.05	93.47	96.42	91.05	97.68	95.79	95.47	99.26
Semeion	91.42	91.60	91.70	94.56	90.53	94.34	95.31	94.65
Madelon	74.29	74.52	79.37	80.38	88.85	82.25	82.54	89.29
Isolet	84.34	85.02	86.98	83.22	92.99	89.16	88.26	93.99
Lung	89.80	92.12	91.20	86.80	89.50	91.09	92.21	95.38
MLL	90.01	89.11	88.93	89.38	86.23	83.70	91.80	93.25
Ovarian	86.38	89.71	86.98	89.56	90.81	92.01	91.23	97.79
CNS	85.38	88.13	89.12	87.30	88.03	84.98	86.89	90.50
Mean Rank	5.07	5.11	4.26	4.70	4.70	4.63	4.59	1.44
Final Rank	7	8	2	5	5	4	3	1

**Table A6:** Feature subset size on FS problem.

Datasets	COA	FATA	HOA	MCOA	MSAACO	SBOA	PLO	ABCCOA
Aggregation	2.00	2.00	2.00	2.00	2.00	2.00	2.00	2.00
Banana	2.00	2.00	2.00	2.00	2.00	2.00	2.00	2.00
Iris	1.00	2.20	1.00	2.00	1.90	3.00	1.00	1.00
Bupa	3.20	2.90	2.10	2.00	2.20	3.00	3.00	3.00
Glass	3.20	3.00	3.00	3.60	4.10	3.60	2.70	3.20
Breastcancer	3.10	2.80	2.80	3.00	2.00	3.00	2.80	2.20
Lipid	1.60	2.60	2.20	2.00	2.40	2.40	1.80	2.00
HeartEW	3.20	4.30	2.60	4.00	3.70	4.70	3.80	5.00
Zoo	5.60	5.90	6.50	4.50	5.30	4.80	6.00	5.60
Vote	1.90	4.10	4.30	2.30	3.30	5.00	3.30	1.00
Congress	1.00	3.70	2.00	1.00	1.80	1.00	4.90	2.30
Lymphography	5.90	5.80	6.50	5.00	5.40	6.30	5.10	5.40
Vehicle	6.50	5.90	6.30	5.70	6.20	7.10	6.60	6.30
WDBC	3.80	6.60	5.40	2.80	3.10	5.60	3.70	4.70
BreastEW	7.60	8.60	11.10	6.40	6.10	9.40	8.00	9.20
SonarEW	19.90	12.30	34.90	13.30	10.80	21.90	17.60	12.20
Libras	21.70	18.30	52.60	14.90	14.80	34.80	32.10	16.90
Hillvalley	16.60	11.00	55.30	15.20	19.20	43.40	40.80	8.80
Musk	36.20	38.70	64.80	29.70	34.40	72.30	73.10	108.00
Clean	13.40	54.10	103.20	38.00	38.60	67.80	70.00	34.50
Semeion	116.60	100.20	163.80	83.50	109.70	118.00	116.80	80.80
Madelon	183.60	161.50	326.10	123.40	104.20	247.30	234.60	61.80
Isolet	194.10	167.20	412.10	152.20	138.70	302.20	289.80	131.70
Lung	138.2	98.3	187.6	387.8	30.9	210.6	56.8	19.43
MLL	73.4	198.2	137.8	102.5	88.4	49.8	56.3	30.8
Ovarian	98.2	70.38	89.8	83.2	76.4	87.6	33.5	17.8
CNS	93.2	74.8	101.5	81.2	94.2	78.2	59.3	26.5
Mean Rnak	4.04	4.37	5.59	3.11	3.22	5.67	4.15	2.85
Final Rank	4	6	7	2	3	8	5	1

**Table A7:** Runtime on FS problem.

Datasets	COA	FATA	HOA	MCOA	MSAACO	SBOA	PLO	ABCCOA
Aggregation	9.62	6.01	7.01	8.45	9.78	8.20	10.02	5.66
Banana	11.09	8.84	9.92	15.21	18.29	22.26	21.92	8.12
Iris	5.49	6.97	6.48	8.74	8.75	9.22	8.97	4.95
Bupa	6.19	7.95	8.70	9.36	8.61	9.40	9.81	5.78
Glass	5.97	8.61	10.26	9.12	9.11	9.43	9.53	5.74
Breastcancer	6.57	9.23	7.36	10.30	9.47	10.30	10.63	6.94
Lipid	6.88	9.49	10.68	9.79	9.02	10.19	10.12	6.28
HeartEW	8.58	9.45	7.06	9.83	9.88	9.75	9.72	6.46
Zoo	8.65	9.26	9.82	9.63	9.49	9.48	9.33	6.41
Vote	8.10	9.47	8.60	10.14	10.07	10.20	9.88	6.28
Congress	7.46	9.55	9.78	9.81	9.98	9.94	10.06	6.03
Lymphography	8.24	9.19	9.94	9.35	9.63	9.87	9.46	6.48
Vehicle	10.62	10.96	8.23	11.52	11.90	11.79	11.81	7.81
WDBC	9.46	9.68	7.40	10.34	13.04	10.01	10.86	6.39
BreastEW	10.15	9.43	9.40	10.99	10.85	11.01	10.93	7.06
SonarEW	8.78	8.43	9.28	9.41	9.01	9.76	9.53	6.59
Libras	9.71	8.72	9.49	10.00	10.17	8.99	11.50	6.93
Hillvalley	9.87	9.27	9.69	10.59	10.81	11.50	12.19	6.42
Musk	11.68	10.34	10.33	10.90	11.20	12.20	12.96	6.55
Clean	10.19	9.72	9.87	11.11	10.17	11.94	12.01	6.59
Semeion	25.24	21.99	22.09	25.53	24.20	27.37	27.87	18.77
Madelon	42.40	57.20	63.49	42.30	43.07	66.32	64.94	38.34
Isolet	26.61	34.41	33.64	31.50	31.37	44.53	41.53	26.89
Lung	159.01	116.91	119.00	105.60	101.40	90.30	89.50	79.29
MLL	198.02	210.98	198.23	141.45	138.12	158.10	110.98	90.18
Ovarian	181.24	178.91	198.22	138.91	145.98	156.32	129.98	101.30
CNS	78.27	86.44	87.68	93.21	101.20	93.03	87.88	69.00
Mean Rnak	3.52	3.59	4.48	5.33	5.37	6.33	6.30	1.07
Final Rank	2	3	4	5	6	8	7	1
